# Arabidopsis ACYL CARRIER PROTEIN4 and RHOMBOID LIKE10 act independently in chloroplast phosphatidate synthesis

**DOI:** 10.1093/plphys/kiad483

**Published:** 2023-09-02

**Authors:** Yang Xu, Shrikaar Kambhampati, Stewart A Morley, Ron Cook, John Froehlich, Doug K Allen, Christoph Benning

**Affiliations:** DOE-Plant Research Laboratory, Michigan State University, East Lansing, MI 48824, USA; Donald Danforth Plant Science Center, St. Louis, MO 63132, USA; Donald Danforth Plant Science Center, St. Louis, MO 63132, USA; United States Department of Agriculture, Agriculture Research Service, St. Louis, MO 63132, USA; DOE-Plant Research Laboratory, Michigan State University, East Lansing, MI 48824, USA; Department of Biochemistry and Molecular Biology, Michigan State University, East Lansing, MI 48824, USA; DOE-Plant Research Laboratory, Michigan State University, East Lansing, MI 48824, USA; Department of Biochemistry and Molecular Biology, Michigan State University, East Lansing, MI 48824, USA; Donald Danforth Plant Science Center, St. Louis, MO 63132, USA; United States Department of Agriculture, Agriculture Research Service, St. Louis, MO 63132, USA; DOE-Plant Research Laboratory, Michigan State University, East Lansing, MI 48824, USA; Department of Biochemistry and Molecular Biology, Michigan State University, East Lansing, MI 48824, USA; Department of Plant Biology, Michigan State University, East Lansing, MI 48824, USA

## Abstract

ACYL CARRIER PROTEIN4 (ACP4) is the most abundant ACP isoform in Arabidopsis (*Arabidopsis thaliana*) leaves and acts as a scaffold for *de novo* fatty acid biosynthesis and as a substrate for acyl-ACP-utilizing enzymes. Recently, ACP4 was found to interact with a protein-designated plastid RHOMBOID LIKE10 (RBL10) that affects chloroplast monogalactosyldiacylglycerol (MGDG) biosynthesis, but the cellular function of this interaction remains to be explored. Here, we generated and characterized *acp4 rbl10* double mutants to explore whether ACP4 and RBL10 directly interact in influencing chloroplast lipid metabolism. Alterations in the content and molecular species of chloroplast lipids such as MGDG and phosphatidylglycerol were observed in the *acp4* and *rbl10* mutants, which are likely associated with the changes in the size and profiles of diacylglycerol (DAG), phosphatidic acid (PA), and acyl-ACP precursor pools. ACP4 contributed to the size and profile of the acyl-ACP pool and interacted with acyl-ACP-utilizing enzymes, as expected for its role in fatty acid biosynthesis and chloroplast lipid assembly. RBL10 appeared to be involved in the conversion of PA to DAG precursors for MGDG biosynthesis as evidenced by the increased 34:*x* PA and decreased 34:*x* DAG in the *rbl10* mutant and the slow turnover of radiolabeled PA in isolated chloroplasts fed with [^14^C] acetate. Interestingly, the impaired PA turnover in *rbl10* was partially reversed in the *acp4 rbl10* double mutant. Collectively, this study shows that ACP4 and RBL10 affect chloroplast lipid biosynthesis by modulating substrate precursor pools and appear to act independently.

## Teaser

Arabidopsis ACYL CARRIER PROTEIN 4 and RHOMBOID LIKE 10 independently affect chloroplast membrane lipid biosynthesis by modulating substrate precursor pools.

## Introduction

Glycerolipids make up the bulk of the lipids of chloroplast membranes, providing essential structural support for the photosynthetic machinery (Botella et al. 2017; Lavell and Benning 2019; Garab et al. 2022). The major glycerolipids in chloroplast membranes include galactolipids, i.e. mono- and digalactosyldiacylglycerol (MGDG and DGDG, which together account for >80% of the total lipids), sulfolipids, i.e. sulfoquinovosyldiacylglycerol (SQDG), and phospholipids, i.e. phosphatidylglycerol (PG) and phosphatidylcholine (PC), the latter of which is primarily found in the outer envelope membrane. In Arabidopsis (*Arabidopsis thaliana*), fatty acids are predominately synthesized *de novo* in the chloroplasts and are either directly incorporated into glycerolipids in the chloroplasts (the chloroplast pathway) or exported to the endoplasmic reticulum (ER) for assembly (the ER pathway) (Chapman and Ohlrogge 2012). Both the chloroplast and ER pathways for glycerolipid assembly start with sequential acylation of the *sn*-1 and 2 positions of glycerol-3-phosphate to yield phosphatidic acid (PA) with the catalysis of glycerol-3-phosphate acyltransferases (GPATs) and lysophosphatidic acid acyltransferases (LPATs) using acyl-acyl carrier protein (acyl-ACP) and acyl-CoA in the chloroplast and ER/cytosol, respectively (Frentzen et al. 1983). Because of the difference in the substrate specificity of LPATs in the respective compartments, glycerolipids from the chloroplast and ER pathways have predominantly C16 or C18 acyl moieties at the *sn*-2 position, respectively (Heinz and Roughan 1983). Chloroplast lipids, particularly MGDG, DGDG, SQDG, and PG, are synthesized predominantly within chloroplasts from PA or PA-derived diacylglycerol (DAG) either from the chloroplast pathway or the ER pathway (Ohlrogge and Browse 1995; Allen et al. 2015). In the latter case, a fraction of the ER assembled glycerolipids is reimported back to chloroplasts for this purpose (Li et al. 2016; Lavell and Benning 2019; Xu et al. 2020). Therefore, the contributions of the 2 pathways to chloroplast lipid biosynthesis can be estimated based on C16 versus C18 ratios of acyl chains. For example, Arabidopsis uses both the chloroplast and ER lipid precursors for MGDG biosynthesis and thus has a specific C16:3/C18:3 ratio of acyl chains in MGDG (Browse et al. 1986).

Acyl carrier proteins (ACPs) are small acidic proteins that act as a carrier of fatty acids for their synthesis up to 18 carbons in length (Alberts et al. 1964; Rock and Cronan 1979). The formed acyl-ACPs serve as substrates for chloroplast enzymes involved in lipid assembly (e.g. GPAT and LPAT), modification (e.g. desaturases), and export (e.g. thioesterases) (Ohlrogge et al. 1979). Arabidopsis has 5 ACP isoforms (ACP1 to 5) in the plastid, and their genes show different expression patterns (Huang et al. 2017). *ACP1* and *ACP5* are predominately expressed in seed and root tissues, respectively, while *ACP2* and *ACP3* are expressed constitutively (Branen et al. 2001; Huang et al. 2017). ACP4 is the most abundant ACP isoform in leaves and appears to have a major role in chloroplast lipid biosynthesis (Branen et al. 2003). Indeed, the *acp4* mutant has lower C16:3 fatty acid content in MGDG (Lavell et al. 2021), but how ACP4 specifically affects chloroplast lipid biosynthesis remains to be explored. Recently, ACP4 was found to form a large protein complex by directly interacting with a putative Arabidopsis plastid rhomboid protease, RHOMBOID LIKE10 (RBL10) (Lavell et al. 2021). RBL10 has previously been found to affect the availability of chloroplast PA precursors for MGDG biosynthesis, and the disruption of *RBL10* leads to a greatly decreased C16:3/C18:3 ratio in MGDG (Lavell et al. 2019), which is similar to but more pronounced than what was observed for the *acp4* mutant. Here, we generated and characterized *acp4 rbl10* double mutants as a means of exploring the roles of ACP4 and its interaction with RBL10 in chloroplast lipid biosynthesis. Our results show that ACP4 and RBL10 act in independent pathways and modulate chloroplast MGDG and PG biosynthesis possibly by affecting the availability and acyl profiles of the lipid precursors.

## Results

### The *acp4 rbl10* mutants are pale green and have altered leaf lipid composition

**Figure 1.**
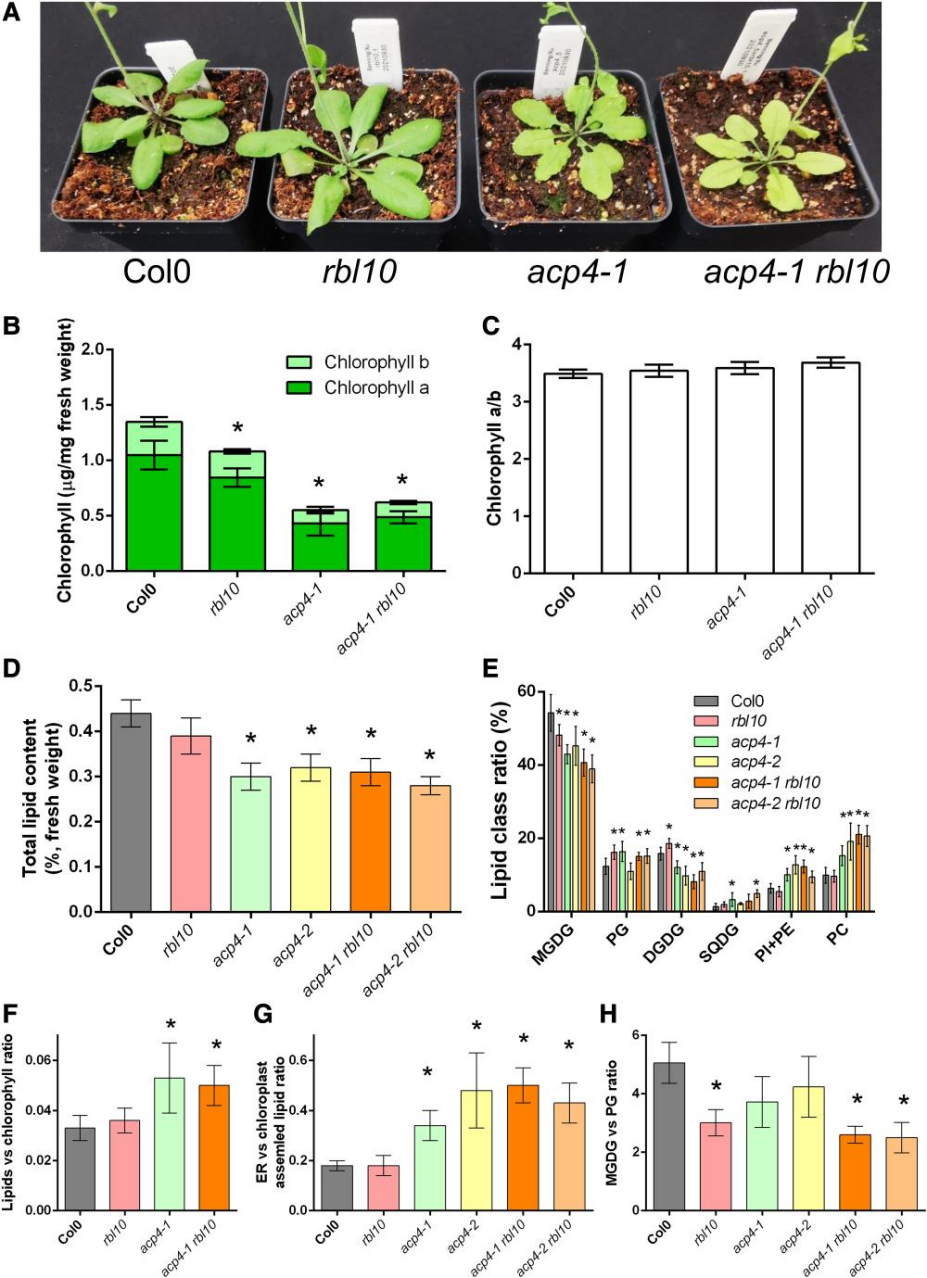
The *acp4* and *rbl10* mutants have reduced total lipid content and altered ratio of lipid classes. **A)** Appearance of the *acp4-1*, *rbl10*, and *acp4-1 rbl10* mutants. **B)** Chlorophyll content. Only one allele (*acp4-1*) was included here and for the chlorophyll-based calculations as the second allele did not differ substantially in appearance and chlorophyll content. **C)** Ratio of chlorophyll a over b. **D)** Total lipid content per fresh weight. **E)** Lipid class ratio. **F)** Normalized lipid content based on chlorophyll content. **G)** Ratio of the ER over chloroplast assembled lipids. **H)** Ratio of MGDG over PG. Data represent the mean ± Sd, *n* = 6 of independent lines. Asterisks indicate significant differences in the lipid content of mutant lines versus the wild-type Col-0 control lines (*t*-test, *P* < 0.05).

Previously, we discovered that RBL10 interacts with lipid proteins such as ACP4 and affects chloroplast lipid biosynthesis (Lavell et al. 2021). To explore the role of this interaction, we crossed the homozygous *acp4* (alleles *acp4-1* and *acp4-2*) and *rbl10* mutant lines to generate double homozygous *acp4 rbl10* mutants. The resulting double homozygous *acp4 rbl10* mutants looked similar to *acp4* mutants (both alleles), which were smaller and had a pale green appearance compared with the wild-type (Col-0) and *rbl10* lines (Fig. 1A). We subsequently focused on the *acp4-1* allele. Chlorophyll analysis confirmed that the *acp4-1* and *acp4-1 rbl10* mutants had a >2-fold decrease in total chlorophyll content based on fresh weight, but the ratio of chlorophyll a over b remained the same compared to Col-0 and *rbl10* lines (Fig. 1, B and C). This suggested that the chlorophyll-containing photosynthetic complexes, including the light harvesting complexes and reaction center complexes (photosystems), were affected at a similar level, decreasing proportionally, in the *acp4-1* and *acp4-1 rbl10* mutants.

The reduction in chlorophyll was associated with the reduced amount of photosynthetic membrane lipids, which decreased by ∼30% on the basis of fresh weight in *acp4* and *acp4 rbl10* mutants (Fig. 1D). However, the *acp4-1* and *acp4-1 rbl10* mutants had increased total lipid level relative to Col-0 and *rbl10*, when normalized to the chlorophyll content and relative lipid class ratios changed in the *acp4*, *rbl10*, and *acp4 rbl10* mutants (Fig. 1, E and F). The *acp4* and *acp4 rbl10* mutants showed increased ratios of lipids found in the ER (PC, phosphatidylethanolamine, and phosphatidylinositol) over chloroplast lipids (MGDG, DGDG, PG, and SQDG) compared to Col-0 and *rbl10* lines (Fig. 1G). The *rbl10* and *acp4 rbl10* mutants had decreased ratios of MGDG over PG relative to Col-0 and *acp4* lines (Fig. 1H).

### ACP4 and RBL10 affect molecular profiles of MGDG and DGDG

**Figure 2.**
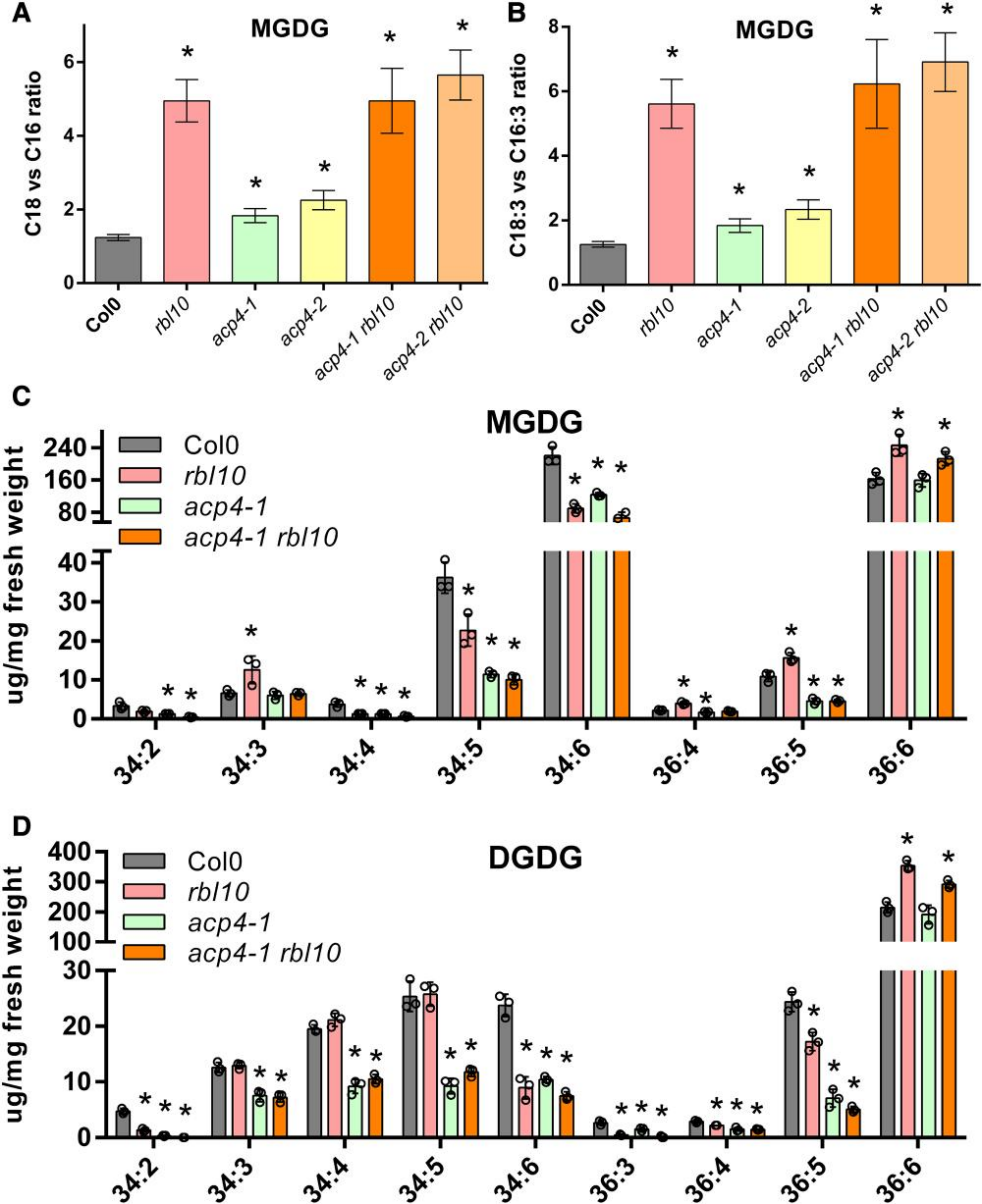
The *acp4* and *rbl10* mutants show altered acyl composition of MGDG and DGDG in leaves. **A)** Ratio of total C18 to C16 fatty acids in MGDG. **B)** Ratio of C18:3 to C16:3 fatty acids in MGDG. **C)** Molecular species and content of MGDG. **D)** Molecular species and content of DGDG. Data represent the mean ± Sd, *n* = 6 of independent lines for **A)** and **B)**, *n* = 3 of independent lines for **C)** and **D)**. Asterisks indicate significant differences in the lipid content of mutant lines versus the wild-type Col-0 control lines (*t*-test, *P* < 0.05).

**Figure 3.**
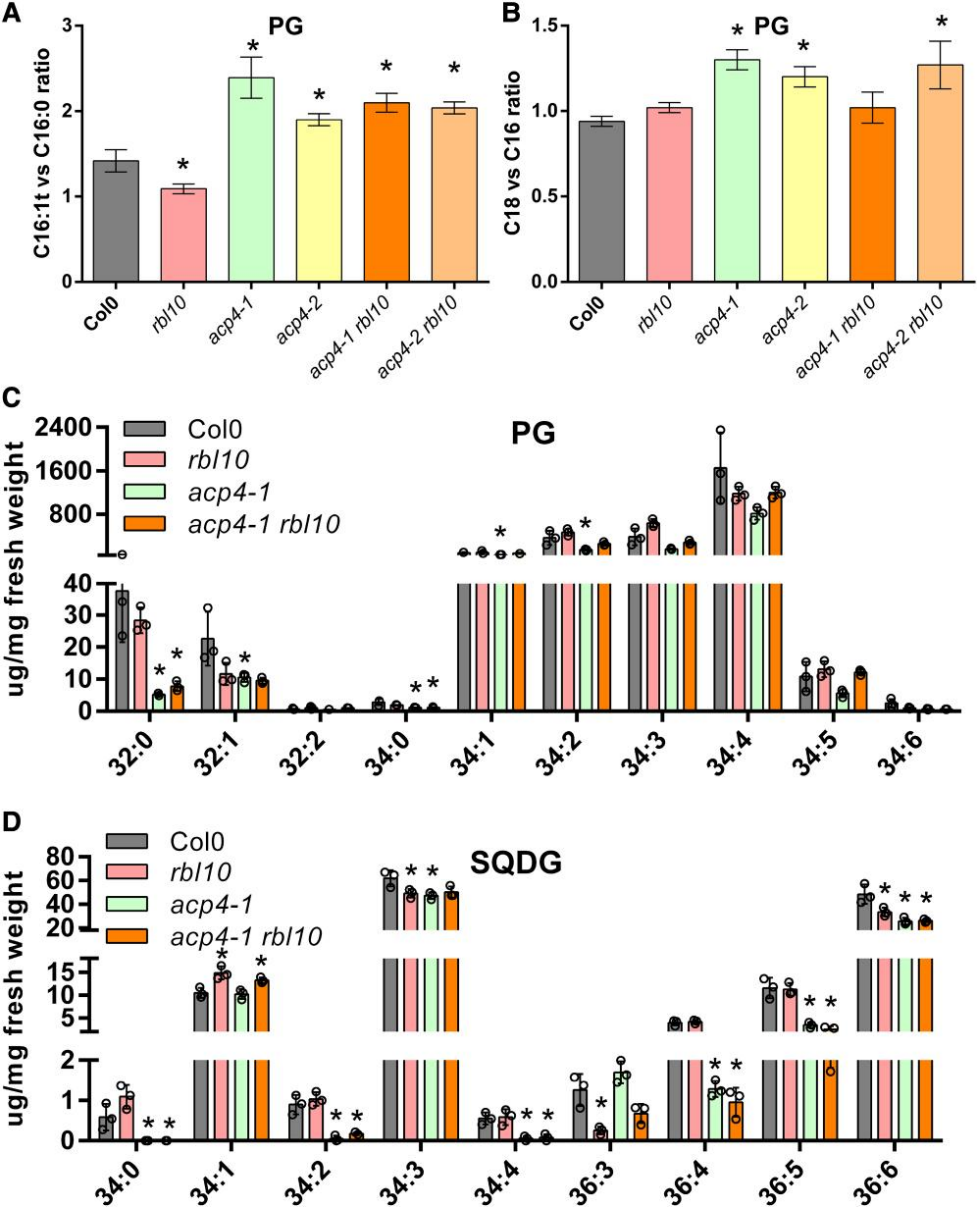
The *acp4* and *rbl10* mutants show altered acyl composition of PG and SQDG in leaves. **A)** Ratio of C16:1t to C16:0 fatty acids in PG. **B)** Ratio of total C18 to C16 fatty acids in PG. **C)** Molecular species and content of PG. **D)** Molecular species and content of SQDG. Data represent the mean ± Sd, *n* = 6 of independent lines for **A)** and **B)**, *n* = 3 of independent lines for **C)** and **D)**. Asterisks indicate significant differences in the lipid content of mutant lines versus the wild-type Col-0 control lines (*t*-test, *P* < 0.05).

Fatty acyl composition analysis of individual lipid classes indicated that *acp4* and *rbl10* affected the acyl composition of different lipid classes, especially the major chloroplast lipids MGDG, DGDG, PG, and SQDG (Figs. 2 and 3; Supplemental Figs. S1 and S2). Consistent with previous observations (Lavell et al. 2021), both the *rbl10* and *acp4* mutants had reduced amounts of C16:3 and increased amounts of C18:3 in MGDG, but the changes in the *acp4* mutants were less obvious (Supplemental Fig. S2). The MGDG acyl composition of the *acp4 rbl10* mutants was identical to that of the *rbl10* mutant, where the ratios of C18:3 over C16:3 fatty acids and total C18 over C16 fatty acids were 5- to 7-fold higher than those of Col-0 (Fig. 2, A and B). The molecular species of MGDG were further explored by lipidomic analysis (Fig. 2C). Together with the fatty acyl composition analysis (Supplemental Fig. S2), the results showed that Arabidopsis leaf MGDG is mainly composed of (in order of decreasing concentration) 36:6 (18:3/18:3), 34:6 (18:3/16:3), 34:5 (18:2/16:3), 36:5 (18:3/18:2), 34:3 (18:3/16:0), and 36:4 (18:3/18:1 or 18:2/18:2). As for the MGDG species in the mutants, the *rbl10* mutant contained more 18:3/18:3 (36:6), 18:3/18:2 (36:5), and 18:3/18:1 (or 18:2/18:2, 36:4) along with less 18:2/16:3 (34:5) and 18:3/16:3 (34:6) than Col-0, whereas the *acp4-1* mutant had similar levels of 18:3/18:3 (36:6) but reduced levels of 18:3/18:2 (36:5), 18:3/16:3 (34:6), and 18:2/16:3 (34:5) relative to Col-0. Apparently, the disruption of *RBL10* affected C16:3 incorporation into MGDG as indicated by the reduced C16:3-containing MGDG, which was compensated by corresponding increases in C18:3-containing MGDG. In contrast, the disruption of *ACP4* decreased chloroplast fatty acid biosynthesis and more strongly affected C16:3 incorporation than C18:3, which led to decreases in most MGDG species, especially C16:3-containing MGDG. One exception was 18:3/18:3 (36:6), which remained at the same level in the *acp4-1* mutant as Col-0. Interestingly, although the MGDG fatty acid profile of the *acp4-1 rbl10* mutant was near-identical to that of the *rbl10* mutant (Supplemental Fig. S2), MGDG species of the double mutant were more like those of the *acp4-1* mutant but with slightly lower 18:3/16:3 (34:6) and higher 18:3/18:3 (36:6). This suggested that although disruption of *ACP4* impaired C16:3 and to a lesser extent C18:3 biosynthesis; further disruption of *RBL10* still led to the shift from 18:3/16:3 (34:6) to 18:3/18:3 (36:6) MGDG, indicating a redirection of lipid precursors derived from the chloroplast pathway to the ER pathway for MGDG biosynthesis.

Evidence for the shift from the chloroplast pathway to the ER pathway extended beyond MGDG to DGDG biosynthesis in the *rbl10* mutants (Fig. 2D; Supplemental Fig. S2). In Arabidopsis leaves, DGDG is mainly synthesized using MGDG derived from the ER pathway and is predominantly composed of 36:6 (18:3/18:3) and to a lesser extent of 34:6 (18:3/16:3), 34:5 (18:2/16:3 or 18:3/16:2), 36:5 (18:3/18:2), 34:4 (18:2/16:2 or 18:1/16:3), and 34:3 (18:3/16:0). Similar to MGDG, disruption of *ACP4* showed no change in the level of 18:3/18:3 DGDG but led to a reduction in all the other DGDG species. Thus, ACP4 may not have a substantial role in the ER pathway for DGDG biosynthesis but likely affects the synthesis of C16 fatty acids and fatty acid desaturation more generally. Disruption of *RBL10* led to increased 18:3/18:3 DGDG at the expense of 18:3/16:3 DGDG, while DGDG species in the *acp4-1 rbl10* double mutant were similar to those in the *acp4-1* mutant but with increased 18:3/18:3. These results suggested a shift from the chloroplast pathway to the ER pathway for DGDG biosynthesis in both of the *rbl10* mutants.

### ACP4 and RBL10 affect the acyl composition of PG and SQDG

MGDG, PG, and SQDG are the major chloroplast lipids synthesized primarily using the chloroplast pathway. Since the chloroplast pathway for galactolipid biosynthesis was affected in the *acp4* and *rbl10* mutants, we inspected the acyl composition and molecular species of PG and SQDG. The acyl composition of PG from the *acp4* and *acp4 rbl10* mutants had significant decreases in C16 groups (especially C16:0) relative to that in non-*acp4* lines (Supplemental Fig. S2). The PG acyl composition of the *acp4 rbl10* mutant was more similar to that of the *acp4* mutant than the *rbl10* mutant, and both the *acp4* and *acp4 rbl10* mutants had increased ratios of C16:1t over C16:0 acyl groups and total C18 over C16 acyl groups (Fig. 3, A and B). In terms of the molecular species, most PG was present in the form of 34:4 (18:3/16:1t), followed by 34:3 (18:2/16:1t or 18:3/16:0), 34:2 (18:1/16:1t or 18:2/16:0), and 34:1 (18:0/16:1t or 18:1/16:0), and a small amount of 32:0 (16:0/16:0) and 32:1 (16:0/16:1t) (Fig. 3C). Although MGDG species from the chloroplast pathway were largely reduced in the *rbl10* mutant, which could affect PG biosynthesis, there were no obvious differences in PG species between Col-0 and *rbl10* plants (Fig. 3C). ACP4 impacted PG biosynthesis more dramatically; there were reduced PG molecular species in the *acp4-1* mutant, especially 32:0 (16:0/16:0), which dropped by ∼90% compared to levels in Col-0, supporting a more specific role for ACP4 in C16:0 biosynthesis. Interestingly, the reduction in 34:4 (18:3/16:1t), 34:3 (18:2/16:1t or 18:3/16:0), and 34:2 (18:1/16:1t or 18:2/16:0) PG in the *acp4-1* mutant was partially restored in the *acp4-1 rbl10* double mutant, probably due to the redirection of the chloroplast C16 acyl groups toward MGDG and the compensation of C18 fatty acids from the ER as a result of blocking *RBL10*.

Unlike the large changes observed for the MGDG and PG profiles of the *acp4* and *rbl10* mutants, the effects of *acp4* and *rbl10* mutations on SQDG biosynthesis were less pronounced in terms of the net content. Col-0 mainly contained (in order of decreasing concentration) 34:3 (18:3/16:0), 36:6 (18:3/18:3), 34:1 (18:1/16:0), 36:5 (18:3/18:2), and 36:4 (18:3/18:1 or 18:2/18:2) SQDG (Fig. 3D; Supplemental Fig. S2). Disruption of *RBL10* had no obvious effect on SQDG, except possibly a small increase in 34:1 with corresponding decreases in 34:3, 36:3, and 36:6. ACP4 more strongly impacted SQDG; disruption of *ACP4* resulted in substantial reductions in 34:3, 36:4, 36:5, 36:6, and other trace SQDG fractions. Further disruption of *RBL10* in *acp4-1* showed no obvious changes in the SQDG profile, supporting that RBL10 might have a limited role in SQDG biosynthesis.

### ACP4 and RBL10 affect the profiles of DAG and PA pools

**Figure 4.**
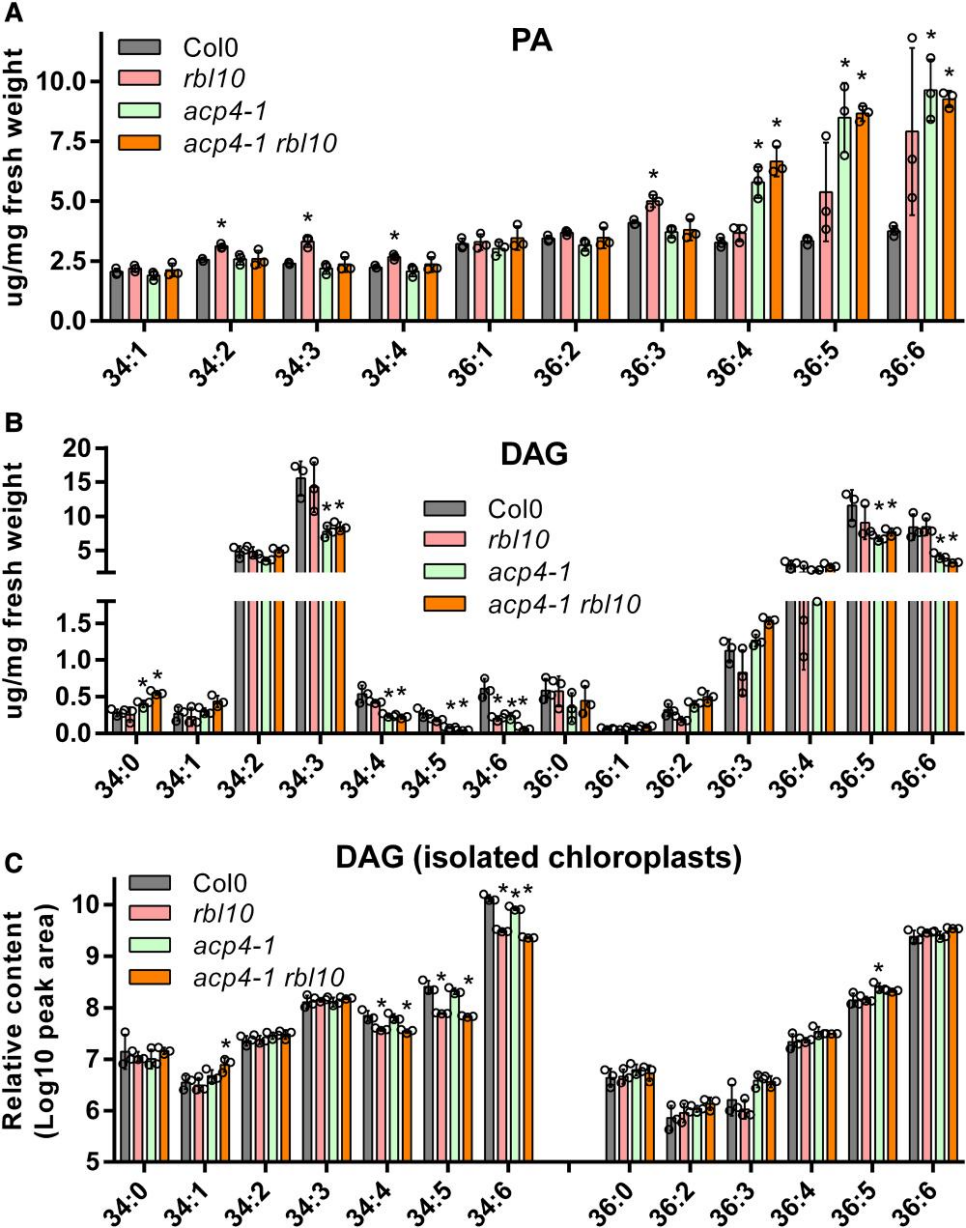
Molecular species of PA and DAG in *acp4* and *rbl10* mutants. **A)** Molecular species and content of PA in leaves. **B)** Molecular species and content of DAG in leaves. **C)** Molecular species and relative content of DAG in isolated chloroplasts. Data represent the mean ± Sd, *n* = 3. Asterisks indicate significant differences in the lipid content of mutant lines versus the wild-type Col-0 control lines (*t*-test, *P* < 0.05).

In chloroplasts, the majority of MGDG (70%), PG (85%), and SQDG (63%) are synthesized using PA or PA-derived DAG from the chloroplast pathway (Browse et al. 1986). PA produced from the catalytic actions of the plastid GPAT (ATS1) and LPAT (ATS2) can be converted to CDP-DAG, which is the precursor for PG biosynthesis. PA can also be dephosphorylated to yield DAG, which can be further converted to MGDG or SQDG. Since PA and the derived DAG are the direct precursors to MGDG, PG, and SQDG, we analyzed their content and molecular species in the leaves and isolated chloroplasts from different Arabidopsis lines (Fig. 4). In leaves, PA and DAG were present at very low levels consisting of 34:*x* (1 to 4) and 36:*x* (1 to 6), and 34:*x* (0 to 6) and 36:*x* (0 to 6), respectively (Fig. 4, A and B). Interestingly, 34:*x* (18:*x*/16:*x*) PAs, such as 34:2, 34:3, and 34:4, were increased in the *rbl10* mutant relative to in Col-0, *acp4-1*, and *acp4-1 rbl10* plants (Fig. 4A). This suggested that RBL10 may restrict the turnover of 34:*x* PA derived from the chloroplast pathway, but that the levels return to Col-0 levels when the production of PA is restricted by further removing *ACP4*. ER-derived 36:*x* (18:*x*/18:*x*) PA was markedly increased in 36:4, 36:5, and 36:6 in the *acp4-1* and *acp4-1 rbl10* mutants and more modestly affected in the *rbl10* mutant (Fig. 4A), indicating a possible compensation of the ER pathway for chloroplast lipid biosynthesis in these mutants. In terms of total leaf DAG, no obvious difference was found in the *rbl10* mutant relative to in Col-0, whereas the *acp4-1* and *acp4-1 rbl10* mutants showed slight decreases in 34:3, 36:5, and 36:6 (Fig. 4B). The decreases in 36:5 and 36:6 DAG likely compensated for the increases in 36:5 and 36:6 PA, possibly caused by the rebalancing of flux between the chloroplast and ER pathways and the rapid turnover of the resulting DAG for chloroplast lipid biosynthesis.

Considering leaves contain mixed sources of PA and DAG from different organelles such as chloroplasts and the ER, intact chloroplasts were isolated and used for the analysis of the chloroplast PA and DAG pools. Unfortunately, PA was not detected in our preparation likely due to its low content and rapid turnover, which may have reflected the activation of PA metabolic enzymes during chloroplast processing. However, DAG could be inspected from isolated chloroplasts and was characterized. The levels of 34:4 (18:3/16:1, 18:2/16:2, 18:1/16:3), 34:5 (18:3/16:2, 18:2/16:3), and 34:6 (18:3/16:3) DAG decreased in the *rbl10* and *acp4-1 rbl10* mutants though not in the *acp4-1* mutant when compared to Col-0 (Fig. 4C). The changes in chloroplast-specific DAG were consistent with the observed decreases in 34:6 (18:3/16:3) MGDG in the *rbl10* and *acp4-1 rbl10* mutants. The decreases in 34:*x* DAG were complemented by the increases in 34:*x* PA in the *rbl10* mutant, indicating that turnover of 34:*x* PA to DAG was blocked by *RBL10* disruption. In the *acp4-1 rbl10* double mutant, 34:*x* DAG decreased without concomitant changes in 34:*x* PA, possibly due to smaller 34:*x* PA pools in the chloroplasts as a result of *ACP4* loss affecting plastid C16 fatty acid biosynthesis. It should be noted that unlike the leaf DAG pools, chloroplast-specific DAG pools showed no obvious change in 36:5 and 36:6 DAG in the *acp4-1* mutants relative to in Col-0, suggesting that observed drops in leaf 36:5 and 36:6 DAG were probably from the ER sources.

### Overexpression of *ACP4* fails to restore the leaf lipid phenotype of *rbl10*

Considering disruption of *RBL10* and *ACP4* has a similar apparent impact on chloroplast lipid composition, we tested whether overexpression of *ACP4* could compensate for the lipid phenotype of *rbl10.* The *ACP4* CDS was expressed under the control of the *Cauliflower mosaic* virus *35S* promoter in both Col-0 and *rbl10* mutant backgrounds, and leaf samples from the obtained T_2_ transgenic lines were used for lipid characterization. Overexpression of *ACP4* in the *rbl10* mutant background failed to restore the leaf lipid phenotype of *rbl10*, leading to no obvious change in the fatty acid composition of total lipids and MGDG (Supplemental Fig. S3). Overexpression of *ACP4* in the Col-0 background, on the other hand, led to slight decreases in C16:3 along with increases in C18:3 in MGDG (Supplemental Fig. S3), which is similar to a prior report on overexpression of *ACP1* in Arabidopsis leaves (Branen et al. 2001). Notably, slight decreases in C16:0 along with increases in C18:3 in PG were observed in *ACP4* overexpressing Col-0 and *rbl10* lines.

### ACP4 affects acyl-ACP profiles and physically interacts with acyl-ACP utilizing enzymes

**Figure 5.**
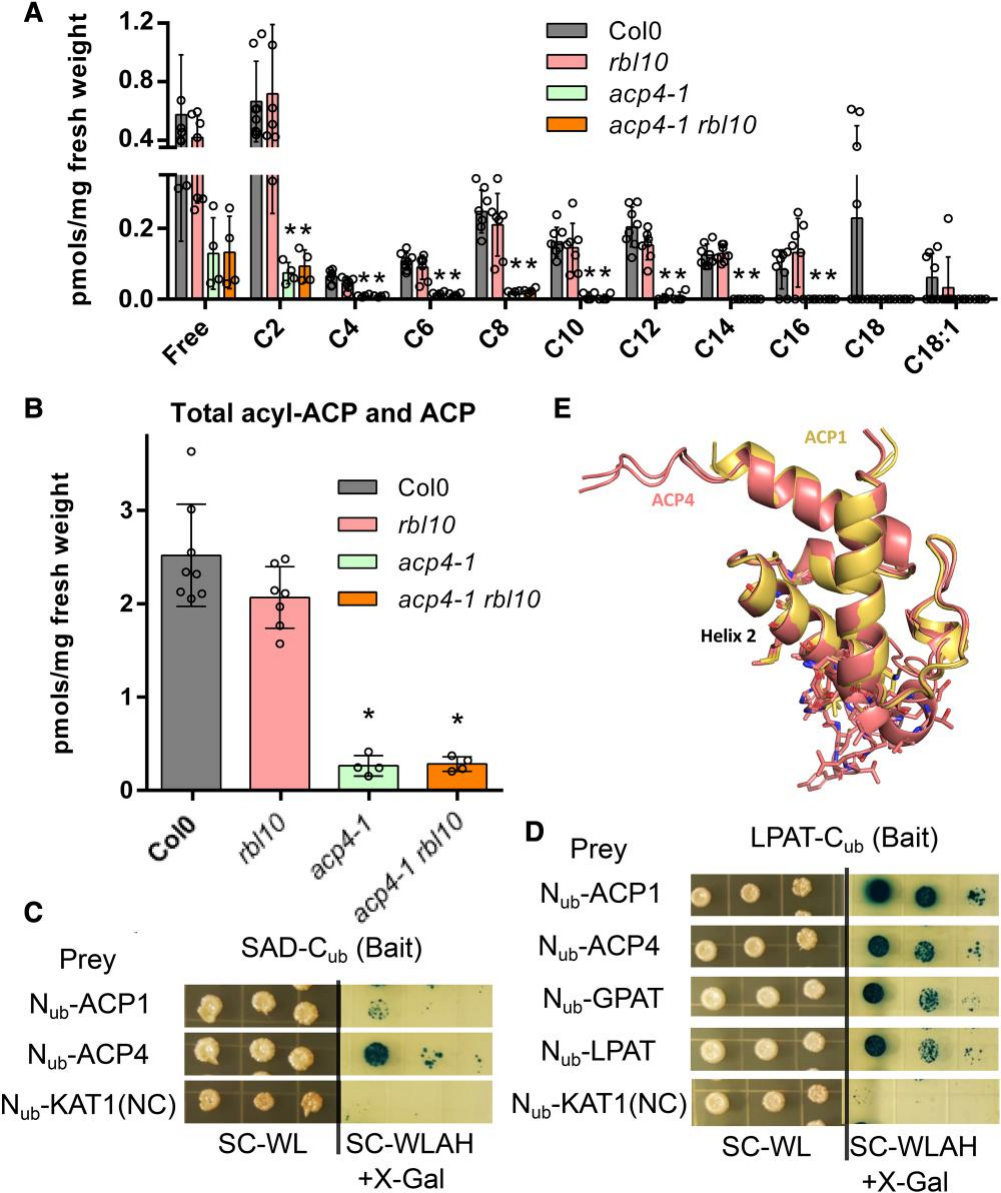
ACP profile in leaves and the possible interactions of ACP with enzymes using acyl-ACPs as substrates. **A)** Acyl-ACP profiles in Arabidopsis leaves. **B)** Total acyl-ACP content in Arabidopsis leaves. **C)** Interaction of SAD with ACP showing by yeast 2-hybrid assay. **D)** Interaction of LPAT with ACP, GPAT, and LPAT showing by yeast 2-hybrid assay. An Arabidopsis K+ channel protein (KAT1) was used as a negative control prey. **E)** Predicted interaction surfaces of ACP1/ACP4 with SAD and LPAT using AlphaFold2. Protein–protein interaction structures between ACP1 and SAD, ACP1 and LPAT, ACP4 and SAD, and ACP4 and LPAT were predicted using AlphaFold2. The structure was superimposed by aligning ACP1 with ACP4. Residues involved in the interactions are shown in stick representation. Data represent the mean ± Sd, *n* = 4 to 8. Asterisks indicate significant differences in the acyl-ACP content of mutant lines versus the wild-type Col-0 control lines (*t*-test, *P* < 0.05). SC-WL, synthetic drop-out agar plates lacking Trp and Leu; SD-WLAH + X-Gal, synthetic drop-out agar plates lacking Trp, Leu, Ade, and His and containing X-Gal.

To further explore the role of ACP4 in plastid lipid biosynthesis, we characterized the acyl-ACP profiles from leaves of Col-0 and the mutants. In Col-0 Arabidopsis leaves, a large portion of ACP is present in the free (apo) form, i.e. not bound to an acyl group, or in the form of acetyl-ACP (C2), while saturated acyl chains ranging from C4, C6 to C18, and C18:1 can be detected under the conditions tested (Fig. 5A). The *rbl10* mutant showed similar content and acyl composition of acyl-ACP to Col-0, whereas the *acp4-1* and *acp4-1 rbl10* mutants had significant decreases in all detected acyl-ACP species, leading to a 90% reduction of the total acyl-ACP content relative to Col-0 (Fig. 5, A and B). Apparently, as the major ACP isoform in Arabidopsis leaves, ACP4 greatly affects the size of the ACP-pools and probably their acyl composition. Therefore, its absence may be limiting and altering acyl lipid biosynthesis in the mutants.

To explore the mechanism by which ACP4 affects PA biosynthesis, we tested the direct interaction of ACP with acyl-ACP utilizing enzymes using a membrane yeast 2-hybrid assay. Indeed, ACP1 and ACP4 were able to interact with stearoyl-ACP desaturase (SAD, Fig. 5C), which converts C16:0/C18:0-ACP to C16:1/C18:1-ACP. ACP1 and ACP4 were also found to directly interact with LPAT when LPAT and ACP were used as a bait and a prey, respectively (Fig. 5D). The structural models of the interaction between ACP1 or 4 and SAD or LPAT were predicted using AlphaFold2 (Bryant et al. 2022), and the predictions indicated that helix 2 of ACP1 and 4 might be involved in their interaction with SAD and LPAT with different confidence levels (Fig. 5E; Supplemental Fig. S4). This is consistent with previous docking and prediction results on the interaction between ACP and fatty acid thioesterase (Blatti et al. 2012). Moreover, we also identified the interaction of LPAT with itself and GPAT (Fig. 5D). GPAT catalyzes the formation of lysophosphatidic acid from glycerol-3-phosphate and acyl-ACP, and lysophosphatidic acid is the substrate for LPAT to further produce PA using C16-ACP as the acyl donor. The identified possible interactions among GPAT, LPAT, and ACP suggested that these lipid enzymes/proteins might form interactomes or metabolomes for local lipid channeling.

### Defects in PA conversion of *rbl10* chloroplasts are compensated by additional inactivation of *ACP4*

**Figure 6.**
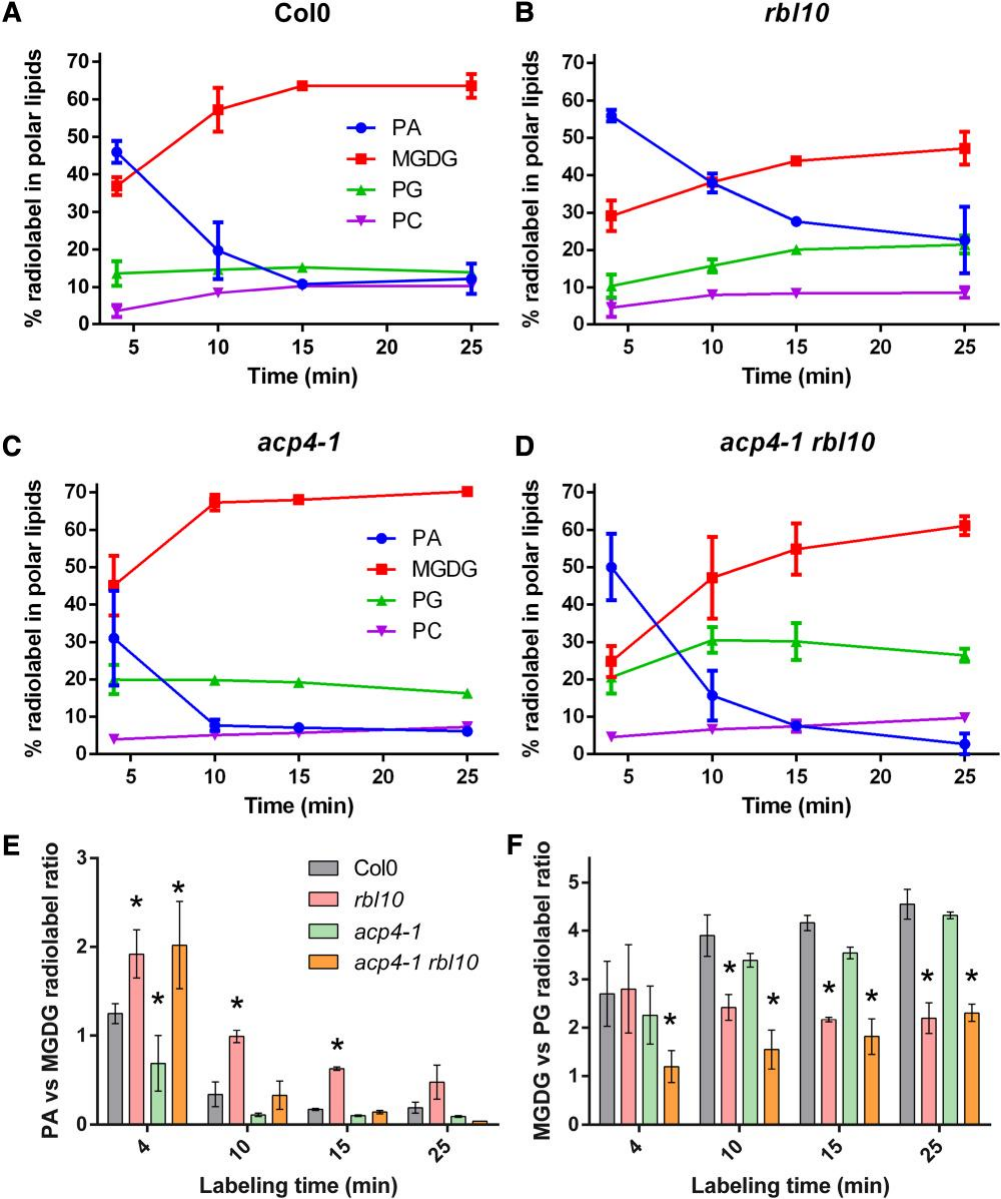
Labeling of isolated intact chloroplasts from the *acp4* and *acp4 rbl10* mutants with acetate [^14^C]. Labeling time course with isolated chloroplasts of wild-type Col-0 **A)**, *rbl10***B)**, *acp4-1***C)**, and *acp4-1 rbl10***D)**. **E)** Ratio of radiolabel in PA to MGDG. **F)** Ratio of radiolabel in MGDG to PG. Data represent the mean ± Sd, *n* = 3. Error bars are only visible if they exceed the diameter of the symbols. Asterisks indicate significant differences between mutant lines and the wild-type Col-0 control lines (*t*-test, *P* < 0.05).

Since the most affected lipids MGDG and PG are mainly synthesized from the chloroplast pathway, intact chloroplasts were isolated from Col-0, *acp4-1*, *rbl10*, and *acp4-1 rbl10* seedlings and fed acetate [^14^C] to probe the carbon flux changes into MGDG and PG. As shown in Fig. 6, the radiolabel was detected in PA, MGDG, PG, and PC across all the assessed chloroplasts, but their relative ratios and changes with time were different. In Col-0 chloroplasts, both PA and MGDG were similarly radiolabeled within 4 min (40 to 50 of total radiolabel %). Subsequently, the radiolabel quickly shifted from PA to MGDG, with MGDG accumulating over 60% of the radiolabel by 25 min, while PA, PG, and PC each accumulated ∼10% (Fig. 6A). In contrast, *rbl10* chloroplasts accumulated 2-fold more radiolabel in PA than MGDG by 4 min, and the shift of radiolabel from PA to MGDG occurred more slowly, with relative label reaching ∼40%, 20%, 20%, and 10% in MGDG, PA, PG, and PC, respectively by 25 min (Fig. 6B). Similar to Col-0, *acp4-1* chloroplasts accumulated radiolabel mainly (>70%) in PA and MGDG at the initial stage, which rapidly shifted from PA to MGDG and was present >60%, <10%, <20%, and <10% in MGDG, PA, PG, and PC at the later stage (Fig. 6C). Although radiolabel accumulated more in PA than MGDG in *acp4-1 rbl10* chloroplasts at the initial stage, conversion of PA to PG and MGDG was more rapid and complete than for *rbl10* chloroplasts at the later stages, where *acp4-1 rbl10* chloroplasts showed ∼60%, 25%, 5%, and 10% in MGDG, PG, PA, and PC, respectively, by 25 min (Fig. 6D). Only *rbl10* chloroplasts accumulated a large amount of radiolabel in PA with the ratio of radiolabel in PA over MGDG >2-fold higher than that in other chloroplasts (Fig. 6E). The *rbl10* and *acp4-1 rbl10* chloroplasts accumulated more radiolabel in PG (20% to 30%) than Col-0 and *acp4-1* chloroplasts; the ratio of radiolabel in MGDG over PG was 2-fold lower in *rbl10* and *acp4-1 rbl10* chloroplasts than in Col-0 and *acp4-1* chloroplasts (Fig. 6F). These results together suggested that the impairment of the conversion of PA to MGDG in *rbl10* chloroplasts was partially compensated for by further disruption of *ACP4*.

## Discussion

In Arabidopsis, chloroplasts are not only the major site of *de novo* fatty acid biosynthesis, but also a major site, in particular the envelope membranes, where these fatty acids are incorporated into PA precursors for the biosynthesis of photosynthetic membrane lipids, including MGDG, PG, and SQDG (Li et al. 2016; Lavell and Benning 2019; Xu et al. 2020). The chloroplast-derived PA is formed by the sequential acylation of glycerol-3-phosphate using acyl-ACPs as acyl donors and is characterized by having a C16 acyl group at the *sn*-2 position due to the unique substrate specificity of chloroplast LPAT (Mongrand et al. 1998; Allen 2016). Previously, the Arabidopsis *rbl10* mutant was found to have a substantial decrease in C16:3 acyl groups in MGDG, which is likely caused by an impairment in transporting PA through the inner envelope membrane or converting it into DAG for MGDG biosynthesis. Recently, we determined that RBL10 directly interacts with lipid metabolism proteins including ACP4 and, hence, forms a protein interactome in chloroplasts, which contributes to chloroplast MGDG biosynthesis. To explore how RBL10 and ACP4 affect chloroplast lipid biosynthesis, we generated *acp4 rbl10* double mutants and *ACP4* overexpression lines and characterized their phenotypes.

### ACP4 and RBL10 act independently, altering photosynthetic membrane characteristics and limiting chloroplast lipid biosynthesis

The *acp4 rbl10* double mutant has similar appearance to the *acp4* mutant, but the lipid phenotype of the *acp4* and *rbl10* mutants is in general an additive in the double mutant (Figs. 1 to 3), which implies that the 2 loci act independently. The double mutant plants were visually similar to the *acp4* mutants, being pale green with reduced chlorophyll content (Fig. 1). These pale green plants (*acp4* and *acp4 rbl10* mutants) also had reduced total lipid content and reduced ratios of chloroplast over ER lipids, but the normalized ratio of lipid over chlorophyll content was slightly higher than that of Col-0 and the *rbl10* mutant (Fig. 1). This may suggest that the pale green plants contain fewer chloroplast membranes and much less chlorophyll. The pale green leaf phenotype of the *acp4* and *acp4 rbl10* mutants resembled several lipid mutants deficient in PG biosynthesis, such as *pgp1*, *ATS2* RNAi downregulation in *ats1-1*, and *cds4 cds5* double mutants (Xu et al. 2002, 2006; Haselier et al. 2010). Indeed, the *acp4* and *acp4 rbl10* mutants had altered fatty acid composition and lipid class ratios, with PG and MGDG being predominantly affected.

The *acp4* and *rbl10* mutants had an increased ratio of total C18 over C16 acyl groups in total lipids, MGDG, and PG compared to Col-0 (Figs. 2 and 3; Supplemental Fig. S1). In terms of MGDG biosynthesis, it appears that disruption of *RBL10* led to an increased reimport of lipids from the ER pathway to the chloroplasts, regardless of whether C16 fatty acid biosynthesis is compromised due to the disruption of *ACP4* or not. As for PG, the *acp4* mutant showed a reduction in 34:*x* PG, which was partially compensated for by further disrupting *RBL10*, probably due to redirection of the plastid C16 fatty acids from MGDG in the *rbl10* mutant. In addition to MGDG and PG, ACP4 and RBL10 affected other chloroplast lipids, such as DGDG and SQDG. Similar to MGDG, DGDG profiles showed a shift from the chloroplast pathway to the ER pathway in the *rbl10* and *acp4* mutants, which is likely a direct result of the profile changes in MGDG, the precursor for DGDG biosynthesis (Fig. 2D; Supplemental Fig. S2). Negligible changes in SQDG profiles were found in the *rbl10* mutants, likely suggesting a limited impact of RBL10 on SQDG biosynthesis (Fig. 3D; Supplemental Fig. S2). This might be related to the distinct sites of MGDG and SQDG synthesis in the chloroplasts, where SQDG is synthesized on the inside of the inner envelope membrane, while the MGDG synthase is active on the outside leaflet of the inner envelope membrane, despite the fact that both MGDG and SQDG are mainly synthesized using DAG derived from the chloroplast pathway. The profiles of different phospholipids were also affected to different extents in the *rbl10* and *acp4* mutants (Supplemental Fig. S5). More profoundly, shifting from 34:*x* PC to 36:*x* PC was observed in the *acp4-1* and *acp4-1 rbl10* mutants, supporting a more specific role of ACP4 in C16 fatty acid biosynthesis.

### ACP4 affects PA and DAG lipid precursor pools by modulating the chloroplast acyl-ACP pool

ACP4 is the major fatty acid carrier in leaf chloroplasts, which provides acyl substrates for chloroplast lipid biosynthesis. Disruption of *ACP4* strongly affected the size, acyl composition, and ACP profile of acyl-ACP pools (Fig. 5), which subsequently is expected to influence the biosynthesis of lipid precursors (e.g. PA and DAG) for MGDG and PG biosynthesis (Fig. 4). The *acp4* and *acp4 rbl10* mutants had reduced fatty acid content (Fig. 1) and reduced acyl-ACP pool size (Fig. 5B). Moreover, disruption of *ACP4* largely altered the apparent acyl composition of the acyl-ACP pool such that quantifiable levels of acyl-ACPs could not be obtained for acyl chains longer than C10 in the *acp4-1* and *acp4-1 rbl10* mutants under our conditions (Fig. 5A). The decrease in acyl-ACPs might indicate that ACP4 plays a larger role as a substrate for the fatty acid synthesis cycle than ACP2-3 and that the latter could be preferred by β-ketoacyl-ACP synthase (KAS) II. Malonyl transacylase establishes the ACP used for each round of fatty acid synthase (FAS) and therefore likely does not discriminate between ACP isoforms to accommodate the different KAS enzymes (Guerra et al. 1986). Whether KAS enzymes are more specific for ACP isoforms, similar to their preferences for acyl chains, is unknown. The lack of available ACP4 would contribute to the overall reduction in total lipids (Fig. 1) and reduced acyl-ACP levels (Fig. 5), with ACP2 to 3 used more substantially in aspects of FAS and resulting in depletion of measurable acyl-ACP chains.

Further, when *ACP4* was overexpressed in the absence of *RBL10* (Supplemental Fig. S3), there was little effect as the ACP4 is incapable of addressing the blocked synthesis of MGDG by the plastid pathway. When *ACP4* was overexpressed alone, MGDG contained more modestly increased amounts of C18:3 and decreased levels of C16:3 fatty acids. This is consistent with the importance of ACP4 in the production of fatty acids through the fatty acid biosynthetic cycle for both the ER and chloroplast pathways; however, the relative drop in C16:3 was not expected and may indicate that competition for acyl chains connected to ACP4 still favors thioesterase or desaturase over acyltransferase activity in the chloroplast. Considering the altered acyl group profile of MGDG and PG in the *acp4* mutant and *ACP4* overexpression lines, i.e. slightly increased C18:3 in MGDG and PG (Supplemental Figs. S2 and S3), it is possible that both deviations from wild-type pool sizes, low and high, facilitate their subsequent elongation and desaturation, leading to more acyl-ACP substrates going to completion.

The contribution of ACP4 to the acyl-ACP pool likely affects the content and acyl composition of chloroplast PA and DAG lipid precursors for MGDG and PG biosynthesis. Previously, the activities of the ER-located DAG acyltransferases have been found to be modulated by acyl-CoA concentrations (Xu et al. 2017). Therefore, it is possible that the acyl-ACP concentration and composition may affect the activity of acyl-ACP dependent enzymes, such as GPAT and LPAT, and thus chloroplast PA biosynthesis. ACP4 seems to affect the content of C16 fatty acids in MGDG and PG (Figs. 2 and 3), which are mainly present at the *sn*-2 position of these lipids by the catalytic action of the C16-selective LPAT. Disruption of *ACP4* led to an increase in the ratio of total C18 over C16 fatty acids in PA (Fig. 4A), which may be favored for further conversion to MGDG and PG. Furthermore, alterations in the ACP isoform concentration caused by the disruption of *ACP4* may also affect lipid precursor availability for chloroplast lipid biosynthesis since the activities of enzymes in the MGDG and PG pathways may possibly be modulated by ACP isoform concentrations considering the direct interaction of ACP with acyl-ACP utilizing enzymes (Fig. 5).

### ACP4 may be part of a metabolon that modulates chloroplast lipid assembly

Since overexpression of *ACP4* and the seed-specific *ACP1* in Arabidopsis leaf tissues resulted in reduced C16:3 content and increased C18:3 content (Supplemental Fig. S3) (Branen et al. 2001), it is possible that the abundance of ACP (apo and acyl-ACP forms) regulates chloroplast LPAT activity and thus modulates chloroplast PA biosynthesis. Acyl-ACP4 may be a more highly favored substrate for chloroplast LPAT and more strongly contribute to chloroplast PA biosynthesis. ACP was largely present in the apo form (Fig. 5A), which may regulate the chloroplast pathway for lipid biosynthesis through interactions with downstream enzymes such as LPAT (Fig. 5, C and D), e.g. acting as a competing substrate for acyl-ACPs or by directly modulating enzyme activities. Moreover, together with our previous findings that ACP4 interacts with RBL10 possibly to facilitate its association with the membrane and availability to LPAT (Lavell et al. 2021), protein–protein interactions of acyl-ACP4 with LPAT and other enzymes and interactions between LPAT and GPAT may have an important role in channeling substrates for chloroplast lipid biosynthesis (Löhden and Frentzen 1988). Structural studies of the *Escherichia coli* type II FAS complex have shown that physical interactions between acyl-ACP and the FAS component enzymes are important for recognition of substrate chain lengths (Bartholow et al. 2021; Sztain et al. 2021). Furthermore, the physical interaction between acyl-ACP and thioesterases in microalgae has also been demonstrated to be crucial for thioesterase substrate preference (Blatti et al. 2012). Increasing evidence supports the presence of lipid biosynthetic interactomes and their importance in modulating lipid metabolism, though further research is needed to elucidate the nature and mechanisms of this noncatalytic regulation of lipid metabolism (Xu et al. 2019; Busta et al. 2022; Xu et al. 2023).

Chloroplast MGDG, PG, and SQDG are predominantly synthesized using PA or PA-derived DAG from the chloroplast pathway, but whether they share the same or different PA or PA-derived DAG pools is not clear. The lipid characterization of *rbl10* and *acp4* mutant lines supports the idea that PG, MGDG, and SQDG biosynthesis may use separate chloroplast PA/DAG precursor pools. Disruption of *RBL10* led to big changes in molecular species of MGDG likely as a result of altered PA and DAG pools. But, the increases in 34:*x* PA and decreases in 34:*x* DAG in the *rbl10* mutant led to no large changes in PG and SQDG, suggesting that different PA and DAG pools might be present for PG and SQDG biosynthesis. ACP4 affects the biosynthesis of fatty acids and their assembly into chloroplast PA and the subsequent PA-derived DAG, and thus its disruption is related to MGDG, PG, and SQDG. Further disruption of *RBL10* partially compensates the reduction of 34:*x* PG in the *acp4* mutant but led to no obvious change in SQDG, probably indicating that the PA used for PG biosynthesis might be different from the PA that gives rise to the DAG precursor for SQDG biosynthesis. Indeed, the Arabidopsis *ats1* mutant deficient in GPAT had abolished chloroplast MGDG biosynthesis, but PG biosynthesis only suffered a slight reduction (10% to 25%) (Xu et al. 2006), supporting the possibility of separate substrate pools for MGDG and PG biosynthesis. In addition, the presence of different DAG pools in the ER for glycerol lipid biosynthesis has been shown (Regmi et al. 2020).

### RBL10 affects the turnover of PA, which can be partially compensated by removing *ACP4*

RBL10 affects the biosynthesis of MGDG derived from the chloroplast pathway, but the direct biochemical activity of RBL10, either proteolytic or otherwise, remains elusive. It is likely that RBL10 is involved in the turnover of 34:*x* PA derived from the plastid pathway to 34:*x* DAG and the subsequent MGDG. This is supported by the lipidomic data showing that the *rbl10* mutants contain higher 34:*x* PA and lower 34:*x* DAG relative to Col-0 (Fig. 4A) and the overaccumulation of radioactivity in PA observed in the [^14^C] acetate feeding assays using isolated *rbl10* chloroplasts (Fig. 6). Interestingly, the defect in PA turnover in the *rbl10* mutant was partially compensated for in the *acp4-1 rbl10* double mutant (Figs. 4 and 6), which might be related to the altered PA pools, e.g. increases in 36:*x* PA, when *ACP4* is disrupted (Fig. 4A). One possibility is that disruption of *ACP4* influences the biosynthesis of chloroplast lipid precursors (e.g. PA) for MGDG and PG biosynthesis by affecting acyl-ACP pools. PA is an allosteric activator of MONOGALACTOSYLDIACYLGLYCEROL SYNTHASE 1 (MGD1), which catalyzes the formation of MGDG, and the acyl composition of PA has different efficiency in activating MGD1, whereby 16:0/16:0-PA is less effective to activate MGD1 than other PA species such as 16:0/18:1-PA (Dubots et al. 2010; Rocha et al. 2013). The alterations in chloroplast PA pool size and acyl composition in the *acp4-1* and *acp4-1 rbl10* mutants (Fig. 4A), therefore, may lead to differential activation of enzymes in MGDG and PG biosynthetic pathways, resulting in a rapid turnover of PA (Figs. 4 and 6). In addition, since MGD1 can also be activated by PG (Dubots et al. 2010), it is possible that the changes in PG acyl composition in the *acp4* and *acp4 rbl10* mutants (Fig. 3) may affect the activation of MGD1.

**Figure 7.**
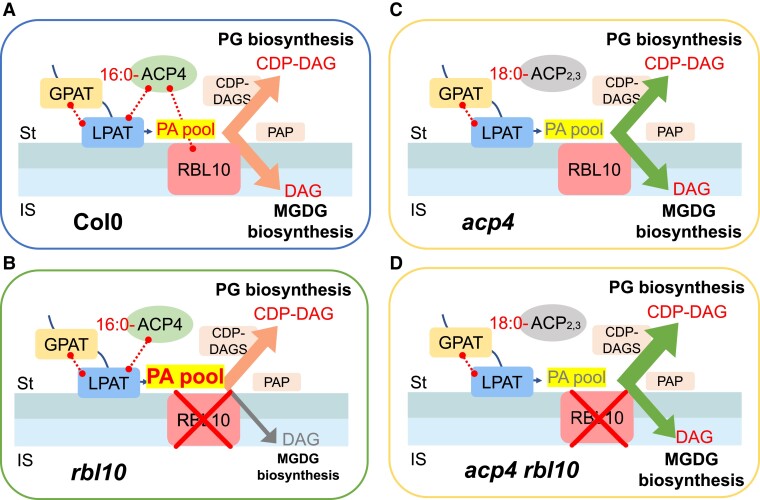
Proposed role of ACP4 and RBL10 in affecting PG and MGDG biosynthesis. In the wild-type Col-0 Arabidopsis **A)**, the interaction of ACP4 with RBL10, GPAT, and LPAT may form local interactomes to facilitate the formation of C16:0-PA precursors for MGDG and PG biosynthesis. In the *rbl10* mutant **B)**, C16:0-PA conversion to MGDG is affected, leading to slow turnover of C16:0-PA and reduced production of C16-MGDG, while PG biosynthesis is not largely affected. In the *acp4* mutant **C)**, the absence of *ACP4* leads to changes in the size, acyl composition, and ACP isoform profile of acyl-ACP pools, and as a result, the size and acyl composition of PA pools. This, in turn, affects the acyl flux to MGDG and PG biosynthesis (indicated by a different arrow color). In the *acp4 rbl10* double mutant **D)**, the slow turnover of PA observed in *rbl10* is partially restored probably because alterations in PA pool size and acyl composition by *ACP4* knockout improve turnover efficiency of PA to MGDG and PG. Observed physical interactions by the membrane yeast 2-hybrid assay are indicated by dashed lines. ST, stroma; IS intermembrane space.

Therefore, we hypothesize that ACP4 and RBL10 modulate plastid MGDG and PG biosynthesis by changing acyl-ACP and PA pools (Fig. 7). In the wild-type Col-0 Arabidopsis, the presence of ACP4 and RBL10 and the interactions of ACP4, GPAT, LPAT, and RBL10 may facilitate the formation of C16:0-PA precursors and chloroplast MGDG and PG biosynthesis. When RBL10 is absent, C16:0-PA conversion to MGDG seems to be affected by the deficiency in the turnover of C16:0-PA or the deficiency in flipping PA to the other side of the inner envelope membrane or other mechanisms (Lavell et al. 2021). When ACP4 is absent, the size, acyl composition, and ACP isoform profile of acyl-ACP pools are altered (Fig. 5), affecting the content and acyl composition of PA precursors (Fig. 4A). When both RBL10 and ACP4 are absent, the alterations in PA pool size and acyl composition may lead to improved turnover efficiency of PA to MGDG and PG.

## Conclusion

Our characterization of *acp4* and *rbl10* single and double mutants showed that ACP4 and RBL10 operate in separate pathways to modulate chloroplast MGDG and PG biosynthesis. Loss of *ACP4* impacts the size, acyl composition, and ACP isoform profile of the acyl-ACP pool and thus influences the chloroplast PA pools for MGDG and PG biosynthesis. RBL10 appears to affect the conversion of chloroplast PA to DAG and then MGDG, but the defects in PA turnover in the *rbl10* mutant can be partially reversed by inactivating *ACP4*, which likely results from altered PA pool content and acyl composition. Taken together, these observations paint a more complex picture of chloroplast lipid biosynthesis with potential regulatory points at multiple levels affecting the size, composition, and location of lipid precursor pools, which likely involves a direct protein–protein interaction network.

## Materials and methods

### Plant materials and growing conditions

The Arabidopsis (*A. thaliana*) T-DNA lines *rbl10*, *acp4-1*, and *acp4-2* were obtained from the Arabidopsis Biological Resource Center with the accession numbers of SALK_036100, SALK_099519C, and SAIL_104_H07 (Alonso et al. 2003), respectively. The double homozygous *acp4-1 rbl10* and *acp4-2 rbl10* plants were obtained by crossing homozygous single mutants. All lines were genotyped for homozygosity. Arabidopsis seeds were grown on MS agar-solidified medium (at 22 °C) or directly in soil (at 22/18 °C, day/night) under 100 to 120 *μ*mol m^−2^ s^−1^ of light in a 16 h light/8 h dark cycle.

### Construct preparation and plant transformation

The coding sequence of *AtACP4* was amplified from Arabidopsis cDNA and inserted down-stream of the constitutive *CaMV 35S* promoter into the *pEARLEYGATE101* vector. The binary vector was introduced into *Agrobacterium tumefaciens* strain GV3101, which was then used to transform Arabidopsis Col-0 and the *rbl10* homozygous mutant lines using the floral dip method (Zhang et al. 2006).

### Chlorophyll quantification

Chlorophyll was extracted from leaf samples using 80% (v/v) acetone, and the chlorophyll content was calculated from the absorbances at 646 and 663 nm according to Porra et al. (1989).

### Intact chloroplast isolation

Intact chloroplasts were isolated from 2- to 3-wk-old Arabidopsis seedlings grown on MS agar-solidified medium as previously described (Inoue et al. 2011; Lavell et al. 2019). In brief, plant tissues were homogenized in a buffer (pH 7.5) containing 330 mm sorbitol, 50 mm HEPES, 1 mm MgCl_2_,1 mm MnCl_2_, 2 mm EDTA, 0.1% (w/v) BSA, and 100 mm ascorbic acid and filtered through double layers of miracloth. Total chloroplasts were pelleted at 1,000 × *g* from the homogenate and were loaded on a Percoll step gradient (40% and 85%, v/v). Intact chloroplasts were collected at the 40%/85% Percoll interface after centrifugation at 2,000 × *g* for 15 min without braking and were washed twice in the above-noted buffer containing no BSA and ascorbic acid.

### Leaf lipid analysis using gas chromatography

For lipid class analysis, total lipids were extracted from 5- to 6-wk-old Arabidopsis rosette leaves using methanol/chloroform/formic acid (20:10:1, v/v/v) as previously described (Wang and Benning 2011). The extracted lipids were separated on a TLC plate (0.25 mm Silica 60 gel, Supelco) treated with (NH_4_)_2_SO_4_ using acetone/toluene/water (91:30:7, v/v/v) as running solvent. Lipids were visualized with brief iodine vapor staining, and the lipid silica spots were scraped from the plate and transmethylated for gas chromatography-flame Ionization detection analysis (Wang and Benning 2011). Fatty acid composition is shown as a mole percentage of total fatty methyl esters, and the ratio of individual lipid classes is presented as a relative mole ratio.

### Lipidomic analysis of leaf and isolated intact chloroplast samples

Leaf lipid extraction was performed using a method previously described (Shiva et al. 2018) with slight modifications. Briefly, 80 to 100 mg of fresh leaf tissues from 5- to 6-wk-old Arabidopsis plants were collected in isopropanol containing 0.01% (w/v) BHT and Ultimate SPLASH ONE mixture (Avanti polar lipids, Birmingham, AL, USA) as internal standards and maintained at 75 °C for 20 min. Lipids were extracted using 2.4 mL of 55:41.5:3.5 (v/v/v) chloroform:methanol:water mixture over a 24 h period, centrifuged at the max speed for 5 min. The supernatant was transferred to a fresh tube, dried under N_2_, and resuspended in 200 *μ*L of 2:2:1 (v/v/v) acetonitrile:isopropanol:chloroform prior to liquid chromatography tandem MS (LC-MS/MS) based lipidomic analyses. Intact chloroplasts were also extracted for lipids using the same method with ∼300 to 400 *μ*g of chlorophyll equivalents containing 17:0/17:0 PA and C15:0 free fatty acid as internal standards.

Lipidomics analysis was performed using an Eksigent Ekspert microLC 200-chromatography system and a CTC analytics Leap HTS PAL liquid handler hooked to a benchtop Q-Exactive Orbitrap MS. Separation was achieved using a custom-made C8 column (100 × 0.5 × 1.7 *µ*m) from Higgins Analytical Inc. (Mountain View, CA, USA) with the mobile phases: 1% 1 m ammonium acetate, 0.1% acetic acid in ddH_2_O (solvent A) and 1% 1 m ammonium acetate, 0.1% acetic acid in 7:3 (v/v) acetonitrile:isopropanol (solvent B), and a flow rate of 40 *µ*L/min. The following gradient modified from Hummel et al. (2011) to adapt to micro flow was used: 0 to 1 min at 55% B, 3 min at 75% B, 8 min at 89% B, 10 min at 99% B, 11 min at 99% B, and 12 min at 55% B followed by equilibration up to 18 min.

Data were acquired for mass ranges of 100 to 1200 *m/z* by full MS at 70,000 resolution in both positive and negative ionization modes. The automatic gain control (AGC) and maximum injection time (IT) were 5 × 10^5^ and 100 ms, respectively. The heated electrospray ionization source was operated with sheath gas, 15 arbitrary units; auxiliary gas, 5 arbitrary units; capillary temperature, 250 °C; auxiliary gas heater temperature, 50 °C; and S-lens RF level, 50. The spray voltage was 4.2 and 3.9 kV in positive and negative modes, respectively. One sample in each group was also used for the top 12 data-dependent acquisition experiment in both ionization modes to generate MS/MS datasets for compound identification. These experiments involved a full MS scan at 70,000 resolution, AGC target 5 × 10^5^, maximum IT 100 ms and MS/MS scans at 17,500 resolution, AGC target 5 × 10^4^, maximum IT 50, 2.0 m/z isolation window, stepped collision energy of 15, 25, and 35 eV, intensity threshold 1 × 10^4^ and 15 s dynamic exclusion. The identity of lipid species was confirmed using MS/MS and retention time. Data integration was performed within the quant browser software available with Thermo Xcalibur. Accurate quantification of lipid molecular species was performed using an isotope dilution method with class-specific deuterated internal standards from Ultimate SPLASH ONE. MGDG, DGDG, SQDG, and PA species, for which deuterated internal standards were not available, were quantified using external standard curves generated upon injection of serial dilutions with commercially available standards. Relative peak areas were used for isolated chloroplasts instead of accurate quantities, where extraction losses could not be calculated for individual lipid molecular species.

### Acyl-ACP profile analysis

Acyl-ACPs were measured by following the method described by Nam et al. (2020). Briefly, fresh leaf tissues were harvested from 5- to 6-wk-old Arabidopsis plants and quickly frozen. Frozen tissues were ground to a fine powder using liquid nitrogen and a mortar and pestle and weighed into individual tubes, taking care throughout the weighing process to avoid thawing. The tissue was homogenized in a solution of 5% (w/v) trichloroacetic acid (TCA). Precipitated proteins were pelleted by centrifugation, washed with 1% (w/v) TCA, and resuspended in MOPS buffer (50 mm, pH 7.6). Proteins were precipitated again by adding TCA to a concentration of 10% (w/v), followed by centrifugation and washing of the pellet with 1% (w/v) TCA. Precipitated proteins were then resuspended in 50 *µ*L of MOPS buffer and digested with Endoproteinase AspN (Cat# P3303, MilliporeSigma, Waltham, MA, USA). Digestions were quenched with the addition of 50 *µ*L methanol, centrifuged at 15,000 × *g*, and acyl-ACPs in the supernatant analyzed using an Ultimate 3000 liquid chromatography system connected to a TSQ Altis mass spectrometer from Thermo Scientific (MilliporeSigma, Waltham, MA, USA) (see parameters in Supplemental Method S1) or AB Sciex QTRAP 6500 (Framingham, MA, USA) with settings, production of acyl-ACP standards, and more extensive protocol details as described in Jenkins et al. (2021).

### Acetate [^14^C] feeding assay

Acetate [^14^C] feeding assays were performed using intact chloroplasts with 0.6 mm UDP-galactose and 10 *μ*Ci/mL of [1,2-^14^C]-acetic acid (58 mCi/mmol, American Radiolabeled Chemical, Inc.) under a table-top LED light (100 *µ*mol m^−2^ s^−1^). Aliquots of chloroplasts were collected at different time points, and lipids were extracted for TLC separation as described above. Lipids were scraped from TLC plates, and radioactivity was quantified using a scintillation counter (MicroBeta Trilux, Perkin-Elmer, MA, USA).

### Membrane yeast 2-hybrid assay

The membrane yeast 2-hybrid assay was performed as described previously (Obrdlik et al. 2004; Lavell et al. 2021). In brief, cDNAs (without the predicted transit peptide sequences) encoding AtACP1 (accession number: AT3G05020), AtACP4 (accession number: AT4G25050), AtGPAT (accession number: AT1G32200), AtLPAT (accession number: AT4G30580), and AtSAD (accession number: AT2G43710) were amplified, sequenced, and cloned in vivo into the pNXgate33-3HA prey vector in the yeast strain THY.AP5 (*MAT*α *URA3 leu2 trp1 his3 loxP*::*ade2*) or the pMetYCgate bait vector in the yeast strain THY.AP4 (*MATa ura3 leu2 lexA::lacZ::trp1 lexA::HIS3 lexA::ADE2*). Yeast transformation was performed using the lithium acetate method (Gietz and Schiestl 2007). The THY.AP4 (C_ub_) and THY.AP5 (N_ub_) transformed cells were mixed to mate, which were then selected for diploids by plating on synthetic complete (SC) medium lacking tryptophan and leucine (SC-WL). Diploid cells were assayed for interaction by replica-plating on SC medium lacking tryptophan, leucine, adenine, and histidine and containing 80 mg/L 5-bromo-4-chloro-3-indolyl-β-D-galactopyranoside (X-Gal) and 150 mm methionine (SC-WLAH + X-Gal). K+ TRANSPORTER OF ARABIDOPSIS (KAT1), a K+ channel protein, was used as a negative control prey.

### Accession numbers

Sequence data from this article can be found in the TAIR data libraries under the accession numbers: AT3G05020 (AtACP1), AT4G25050 (AtACP4), AT1G32200 (AtGPAT), AT4G30580 (AtLPAT), AT2G43710 (AtSAD), and AT1G25290 (AtRBL10).

## Supplementary Material

kiad483_Supplementary_Data

## Supplemental data

The following materials are available in the online version of this article.

**Supplemental Method S1.** LC-MS/MS conditions for acyl-ACP detection on TSQ Altis.

**Supplemental Figure S1.** Lipid characterization of leaves from the *acp4*, *rbl10*, and *acp4 rbl10* mutant plants.

**Supplemental Figure S2.** Fatty acid composition of different lipid classes of leaves from the *acp4*, *rbl10*, and *acp4 rbl10* mutant plants.

**Supplemental Figure S3.** Lipid characterization of leaves from the *ACP4* overexpression lines in Col-0 and *rbl10* mutant backgrounds.

**Supplemental Figure S4.** Predicted protein–protein interaction structures of ACP1 or ACP4 with LPAT or SAD using AlphaFold2.

**Supplemental Figure S5.** Molecular species of phospholipids of leaves from the *acp4*, *rbl10*, and *acp4 rbl10* mutant plants.

## Dive Curated Terms

The following phenotypic, genotypic, and functional terms are of significance to the work described in this paper:

ACP4 Gramene: AT4G25050
ACP4 Araport: AT4G25050

ACP1 Gramene: AT3G05020
ACP1 Araport: AT3G05020

ACP2 Gramene: AT1G54580
ACP2 Araport: AT1G54580

ATS1 Gramene: AT1G32200
ATS1 Araport: AT1G32200

PGP1 Gramene: AT2G39290
PGP1 Araport: AT2G39290

ACP3 Gramene: AT1G54630
ACP3 Araport: AT1G54630

ACP5 Gramene: AT5G27200
ACP5 Araport: AT5G27200

MGD1 Gramene: AT4G31780
MGD1 Araport: AT4G31780

RBL10 Gramene: AT1G25290
RBL10 Araport: AT1G25290

ATS2 Gramene: AT4G30580
ATS2 Araport: AT4G30580

## References

[kiad483-B1] Alberts AW , Majerus PW, Talamo B, Vagelos PR. Acyl-carrier protein. II. Intermediary reactions of fatty acid biosynthesis. Biochemistry. 1964:3(10):1563–1571. 10.1021/bi00898a030 14232033

[kiad483-B2] Allen DK . Assessing compartmentalized flux in lipid metabolism with isotopes. Biochim Biophys Acta. 2016:1861(9):1226–1242. 10.1016/j.bbalip.2016.03.017 27003250

[kiad483-B3] Allen DK , Bates PD, Tjellström H. Tracking the metabolic pulse of plant lipid production with isotopic labeling and flux analyses: past, present and future. Prog Lipid Res. 2015:58:97–120. 10.1016/j.plipres.2015.02.002 25773881

[kiad483-B4] Alonso JM , Stepanova AN, Leisse TJ, Kim CJ, Chen H, Shinn P, Stevenson DK, Zimmerman J, Barajas P, Cheuk R, et al Genome-wide insertional mutagenesis of *Arabidopsis thaliana*. Science. 2003:301(5633):653–657. 10.1126/science.1086391 12893945

[kiad483-B5] Bartholow TG , Sztain T, Patel A, Lee DJ, Young MA, Abagyan R, Burkart MD. Elucidation of transient protein-protein interactions within carrier protein-dependent biosynthesis. Commun Biol. 2021:4(1):340. 10.1038/s42003-021-01838-3 33727677 PMC7966745

[kiad483-B6] Blatti JL , Beld J, Behnke C, Mendez M, Mayfield SP, Burkart MD. Manipulating fatty acid biosynthesis in microalgae for biofuel through protein-protein interactions. PLoS One. 2012:7(9):e42949. 10.1371/journal.pone.0042949 23028438 PMC3441505

[kiad483-B7] Botella C , Jouhet J, Block MA. Importance of phosphatidylcholine on the chloroplast surface. Prog Lipid Res. 2017:65:12–23. 10.1016/j.plipres.2016.11.001 27871883

[kiad483-B8] Branen JK , Chiou TJ, Engeseth NJ. Overexpression of *acyl carrier protein-1* alters fatty acid composition of leaf tissue in Arabidopsis. Plant Physiol. 2001:127(1):222–229. 10.1104/pp.127.1.222 11553750 PMC117978

[kiad483-B9] Branen JK , Shintani DK, Engeseth NJ. Expression of antisense *acyl carrier protein-4* reduces lipid content in Arabidopsis leaf tissue. Plant Physiol. 2003:132(2):748–756. 10.1104/pp.102.018622 12805604 PMC167014

[kiad483-B10] Browse J , Warwick N, Somerville CR, Slack CR. Fluxes through the prokaryotic and eukaryotic pathways of lipid synthesis in the “16:3” plant *Arabidopsis thaliana*. Biochem J. 1986:235(1):25–31. 10.1042/bj2350025 3741384 PMC1146643

[kiad483-B11] Bryant P , Pozzati G, Elofsson A. Improved prediction of protein-protein interactions using AlphaFold2. Nat Commun. 2022:13(1):1265. 10.1038/s41467-022-28865-w 35273146 PMC8913741

[kiad483-B12] Busta L , Chapman KD, Cahoon EB. Better together: protein partnerships for lineage- specific oil accumulation. Curr Opin Plant Biol. 2022:66:102191. 10.1016/j.pbi.2022.102191 35220088

[kiad483-B13] Chapman KD , Ohlrogge JB. Compartmentation of triacylglycerol accumulation in plants. J Biol Chem. 2012:287(4):2288–2294. 10.1074/jbc.R111.290072 22090025 PMC3268389

[kiad483-B14] Dubots E , Audry M, Yamaryo Y, Bastien O, Ohta H, Breton C, Maréchal E, Block MA. Activation of the chloroplast monogalactosyldiacylglycerol synthase MGD1 by phosphatidic acid and phosphatidylglycerol. J Biol Chem. 2010:285(9):6003–6011. 10.1074/jbc.M109.071928 20023301 PMC2825394

[kiad483-B15] Frentzen M , Heinz E, McKeon TA, Stumpf PK. Specificities and selectivities of glycerol-3-phosphate acyltransferase and monoacylglycerol-3-phosphate acyltransferase from pea and spinach chloroplasts. Eur J Biochem. 1983:129(3):629–636. 10.1111/j.1432-1033.1983.tb07096.x 6825679

[kiad483-B16] Garab G , Yaguzhinsky LS, Dlouhý O, Nesterov SV, Špunda V, Gasanoff ES. Structural and functional roles of non-bilayer lipid phases of chloroplast thylakoid membranes and mitochondrial inner membranes. Prog Lipid Res. 2022:86:101163. 10.1016/j.plipres.2022.101163 35351472

[kiad483-B17] Gietz RD , Schiestl RH. High-efficiency yeast transformation using the LiAc/SS carrier DNA/PEG method. Nat Protoc. 2007:2(1):31–34. 10.1038/nprot.2007.13 17401334

[kiad483-B18] Guerra DJ , Ohlrogge JB, Frentzen M. Activity of acyl carrier protein isoforms in reactions of plant fatty acid metabolism. Plant Physiol. 1986:82(2):448–453. 10.1104/pp.82.2.448 16665049 PMC1056138

[kiad483-B19] Haselier A , Akbari H, Weth A, Baumgartner W, Frentzen M. Two closely related genes of arabidopsis encode plastidial cytidinediphosphate diacylglycerol synthases essential for photoautotrophic growth. Plant Physiol. 2010:153(3):1372–1384. 10.1104/pp.110.156422 20442275 PMC2899908

[kiad483-B20] Heinz E , Roughan PG. Similarities and differences in lipid metabolism of chloroplasts isolated from 18:3 and 16:3 plants. Plant Physiol. 1983:72(2):273–279. 10.1104/pp.72.2.273 16662992 PMC1066223

[kiad483-B21] Huang J , Xue C, Wang H, Wang L, Schmidt W, Shen R, Lan P. Genes of *ACYL CARRIER PROTEIN* family show different expression profiles and overexpression of *ACYL CARRIER PROTEIN 5* modulates fatty acid composition and enhances salt stress tolerance in Arabidopsis. Front Plant Sci. 2017:8:987. 10.3389/fpls.2017.00987 28642782 PMC5463277

[kiad483-B22] Hummel J , Segu S, Li Y, Irgang S, Jueppner J, Giavalisco P. Ultra performance liquid chromatography and high resolution mass spectrometry for the analysis of plant lipids. Front Plant Sci. 2011:2:54. 10.3389/fpls.2011.00054 22629264 PMC3355513

[kiad483-B23] Inoue H , Wang F, Inaba T, Schnell DJ. Energetic manipulation of chloroplast protein import and the use of chemical cross-linkers to map protein-protein interactions. Methods Mol Biol. 2011:774:307–320. 10.1007/978-1-61779-234-2_18 21822846 PMC4049570

[kiad483-B24] Jenkins LM , Nam J-W, Evans BS, Allen DK. Quantification of acyl-acyl carrier proteins for fatty acid synthesis using LC-MS/MS. Methods Mol Biol. 2021:2295:219–247. 10.1007/978-1-0716-1362-7_13 34047980

[kiad483-B25] Lavell AA , Benning C. Cellular organization and regulation of plant glycerolipid metabolism. Plant Cell Physiol. 2019:60(6):1176–1183. 10.1093/pcp/pcz016 30690552 PMC6553661

[kiad483-B26] Lavell A , Froehlich JE, Baylis O, Rotondo AD, Benning C. A predicted plastid rhomboid protease affects phosphatidic acid metabolism in *Arabidopsis thaliana*. Plant J. 2019:99(5):978–987. 10.1111/tpj.14377 31062431 PMC6711814

[kiad483-B27] Lavell A , Smith M, Xu Y, Froehlich JE, De La Mora C, Benning C. Proteins associated with the *Arabidopsis thaliana* plastid rhomboid like protein RBL10. Plant J. 2021:108(5):1332–1345. 10.1111/tpj.15514 34582071 PMC9219029

[kiad483-B28] Li N , Xu C, Li-Beisson Y, Philippar K. Fatty acid and lipid transport in plant cells. Trends Plant Sci. 2016:21(2):145–158. 10.1016/j.tplants.2015.10.011 26616197

[kiad483-B29] Löhden I , Frentzen M. Role of plastidial acyl-acyl carrier protein: glycerol 3-phosphate acyltransferase and acyl-acyl carrier protein hydrolase in channelling the acyl flux through the prokaryotic and eukaryotic pathway. Planta. 1988:176(4):506–512. 10.1007/BF00397657 24220947

[kiad483-B30] Mongrand S , Bessoule JJ, Cabantous F, Cassagne C. The C16:3/C18:3 fatty acid balance in photosynthetic tissues from 468 plant species. Phytochemistry. 1998:49(4):1049–1064. 10.1016/S0031-9422(98)00243-X

[kiad483-B31] Nam J , Jenkins LM, Li J, Evans BS, Jaworski JG, Allen DK. A general method for quantification and discovery of acyl groups attached to acyl carrier proteins in fatty acid metabolism using LC-MS/MS. Plant Cell. 2020:32(4):820–832. 10.1105/tpc.19.00954 32060179 PMC7145485

[kiad483-B32] Obrdlik P , El-Bakkoury M, Hamacher T, Cappellaro C, Vilarino C, Fleischer C, Ellerbrok H, Kamuzinzi R, Ledent V, Blaudez D, et al K+ channel interactions detected by a genetic system optimized for systematic studies of membrane protein interactions. Proc Natl Acad Sci U S A. 2004:101(33):12242–12247. 10.1073/pnas.0404467101 15299147 PMC514463

[kiad483-B33] Ohlrogge J , Browse J. Lipid biosynthesis. Plant Cell. 1995:7(7):957–970. 10.1105/tpc.7.7.957 7640528 PMC160893

[kiad483-B34] Ohlrogge JB , Kuhn DN, Stumpf PK. Subcellular localization of acyl carrier protein in leaf protoplasts of *Spinacia oleracea*. Proc Natl Acad Sci U S A. 1979:76(3):1194–1198. 10.1073/pnas.76.3.1194 286305 PMC383216

[kiad483-B35] Porra RJ , Thompson WA, Kriedemann PE. Determination of accurate extinction coefficients and simultaneous equations for assaying chlorophylls a and b extracted with four different solvents: verification of the concentration of chlorophyll standards by atomic absorption spectroscopy. Biochim Biophys Acta. 1989:975(3):384–394. 10.1016/S0005-2728(89)80347-0

[kiad483-B36] Regmi A , Shockey J, Kotapati HK, Bates PD. Oil-producing metabolons containing DGAT1 utilize separate substrate pools from those containing DGAT2 or PDAT. Plant Physiol. 2020:184(2):720–737. 10.1104/pp.20.00461 32732347 PMC7536707

[kiad483-B37] Rocha J , Audry M, Pesce G, Chazalet V, Block MA, Maréchal E, Breton C. Revisiting the expression and purification of MGD1, the major galactolipid synthase in Arabidopsis to establish a novel standard for biochemical and structural studies. Biochimie. 2013:95(4):700–708. 10.1016/j.biochi.2012.11.011 23200907

[kiad483-B38] Rock CO , Cronan JE. Re-evaluation of the solution structure of acyl carrier protein. J Biol Chem. 1979:254(19):9778–9785. 10.1016/S0021-9258(19)83584-6 39930

[kiad483-B39] Shiva S , Enninful R, Roth MR, Tamura P, Jagadish K, Welti R. An efficient modified method for plant leaf lipid extraction results in improved recovery of phosphatidic acid. Plant Methods. 2018:14(1):14. 10.1186/s13007-018-0282-y 29449874 PMC5812192

[kiad483-B40] Sztain T , Bartholow TG, Lee DJ, Casalino L, Mitchell A, Young MA, Wang J, McCammon JA, Burkart MD. Decoding allosteric regulation by the acyl carrier protein. Proc Natl Acad Sci U S A. 2021:118(16):e2025597118. 10.1073/pnas.2025597118 33846262 PMC8072227

[kiad483-B41] Wang Z , Benning C. *Arabidopsis thaliana* polar glycerolipid profiling by thin layer chromatography (TLC) coupled with gas-liquid chromatography (GLC). J Vis Exp. 2011:49:e2518. 10.3791/2518 PMC3197303 21445048

[kiad483-B42] Xu Y , Caldo KMP, Jayawardhane K, Ozga JA, Weselake RJ, Chen G. A transferase interactome that may facilitate channeling of polyunsaturated fatty acid moieties from phosphatidylcholine to triacylglycerol. J Biol Chem. 2019:294(41):14838–14844. 10.1074/jbc.AC119.010601 31481466 PMC6791306

[kiad483-B43] Xu Y , Chen G, Greer MS, Caldo KMP, Ramakrishnan G, Shah S, Wu L, Lemieux MJ, Ozga J, Weselake RJ. Multiple mechanisms contribute to increased neutral lipid accumulation in yeast producing recombinant variants of plant diacylglycerol acyltransferase 1. J Biol Chem. 2017:292(43):17819–17831. 10.1074/jbc.M117.811489 28900030 PMC5663881

[kiad483-B44] Xu C , Fan J, Shanklin J. Metabolic and functional connections between cytoplasmic and chloroplast triacylglycerol storage. Prog Lipid Res. 2020:80:101069. 10.1016/j.plipres.2020.101069 33127353

[kiad483-B45] Xu C , Härtel H, Wada H, Hagio M, Yu B, Eakin C, Benning C. The *pgp1* mutant locus of Arabidopsis encodes a phosphatidylglycerolphosphate synthase with impaired activity. Plant Physiol. 2002:129(2):594–604. 10.1104/pp.002725 12068104 PMC161686

[kiad483-B46] Xu Y , Singer SD, Chen G. Protein interactomes for plant lipid biosynthesis and their biotechnological applications. Plant Biotechnol J. 2023:21(9): 1734–1744. 10.1111/pbi.14027 36762506 PMC10440990

[kiad483-B47] Xu C , Yu B, Cornish AJ, Froehlich JE, Benning C. Phosphatidylglycerol biosynthesis in chloroplasts of Arabidopsis mutants deficient in acyl-ACP glycerol-3-phosphate acyltransferase. Plant J. 2006:47(2):296–309. 10.1111/j.1365-313X.2006.02790.x 16774646

[kiad483-B48] Zhang X , Henriques R, Lin S-S, Niu Q-W, Chua N-H. Agrobacterium-mediated transformation of *Arabidopsis thaliana* using the floral dip method. Nat Protoc. 2006:1(2):641–646. 10.1038/nprot.2006.97 17406292

