# Supplementary material for: Arabidopsis ACYL CARRIER PROTEIN4 and RHOMBOID LIKE10 act independently in chloroplast phosphatidate synthesis
Source: Plant Physiol. 2023 Sep 2;193(4):2661–76. doi: 10.1093/plphys/kiad483 (PMC10803724; doi:10.1093/plphys/kiad483)
Supplement: kiad483_Supplementary_Data [file kiad483_supplementary_data.pdf]

Supplemental Data

Supplemental Method S1. LC-MS/MS conditions for acyl-ACP detection on TSQ Altis.

Supplemental Figure S1. Lipid characterization of leaves from the *acp4*, *rbl10*, and *acp4 rbl10* mutant plants.

Supplemental Figure S2. Fatty acid composition of different lipid classes of leaves from the *acp4*, *rbl10*, and *acp4 rbl10* mutant plants.

Supplemental Figure S3. Lipid characterization of leaves from the *ACP4* over-expression lines in Col-0 and *rbl10* mutant backgrounds.

Supplemental Figure S4. Predicted protein–protein interaction structures of ACP1 or ACP4 with LPAT or SAD using AlphaFold2.

Supplemental Figure S5. Molecular species of phospholipids of leaves from the *acp4*, *rbl10*, and *acp4 rbl10* mutant plants.

Supplemental Method S1. LC-MS/MS conditions for acyl-ACP detection on TSQ Altis

Acyl-ACPs were separated on a Discovery BIO Wide Pore C18 column (10cm x 2.1 mm; 3µm, Millipore Sigma) with a Dionex Ultimate 3000 RS and detected by LC-MS/MS using an Thermo Scientific TSQ Altis with Xcalibur software. Buffer A: acetonitrile/10 mM ammonium formate and formic acid, pH 3.5 (10:90, v/v); Buffer B: acetonitrile/10 mM ammonium formate and formic acid, pH 3.5 (90:10, v/v). (For additional details on non-mass spectrometry methodology see Jenkins et al., 2021 referenced in main article).

1. Set flow rate to 0.3 ml/min, 100% buffer A.
2. LC buffer gradient (pictured right): 0-2.5 min, 0-7.5% B; 2.5-5.8 min, 7.5-70% B, 5.8-6.67 min, 70-100% B; 6.67-10.83 min, hold at 100% B; 10.83-11.67 min, 100%-0, 11.67-17 min hold at 0% B, stop at 17 min.
3. MS parameters: positive ion and selected reaction monitoring (SRM) mode; sheath gas, 50; aux gas, 10; sweep gas, 1; ion spray voltage, 3.25 kV; vaporizer temperature, 350°; ion transfer tube temperature, 325°.
4. SRM parameters presented in the table below.

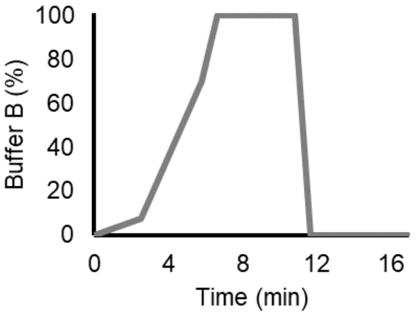

Compound	Start Time (min)	End Time (min)	Precursor (m/z)	Product (m/z)	Collision Energy (V)	Dwell Time (ms)	RF Lens (V)
apo-DSL	0.75	16.67	334.16	203.07	45	12.223	50
apo-DSL 15N	0.75	16.67	337.16	205.07	45	12.223	50
Holo-DSL	1	16.67	674.2	261.1	45	12.223	50
Holo-DSL 15N	1	16.67	677.2	261.1	45	12.223	50
malonyl	1.5	16.67	760.2	347.1	45	12.223	50
malonyl 15N	1.5	16.67	763.2	347.1	45	12.223	50
2(0)	1.5	16.67	716.3	303.2	45	12.223	50
2(0) 15N	1.5	16.67	719.3	303.2	45	12.223	50
4(0)	1.5	16.67	744.3	331.2	45	12.223	50
4(0) 15N	1.5	16.67	747.3	331.2	45	12.223	50
6(0)	1.5	16.67	772.3	359.2	55	12.223	50
6(0) 15N	1.5	16.67	775.3	359.2	55	12.223	50
8(0)	1.5	16.67	800.4	387.3	55	12.223	50
8(0) 15N	1.5	16.67	803.4	387.3	55	12.223	50
10(0)	1.5	16.67	828.4	415.3	55	12.223	50
10(0) 15N	1.5	16.67	831.4	415.3	55	12.223	50
12(0)	1.5	16.67	856.4	443.3	55	12.223	50
12(0) 15N	1.5	16.67	859.4	443.3	55	12.223	50
14(0)	1.5	16.67	884.4	471.3	55	12.223	50
14(0) 15N	1.5	16.67	887.4	471.3	55	12.223	50
16(0)	1.5	16.67	912.5	499.4	55	12.223	50
16(0) 15N	1.5	16.67	915.5	499.4	55	12.223	50
18(0)	1.5	16.67	940.5	527.4	55	12.223	50
18(0) 15N	1.5	16.67	943.5	527.4	55	12.223	50
18(1)	1.5	16.67	938.5	525.4	55	12.223	50
18(1) 15N	1.5	16.67	941.5	525.4	55	12.223	50

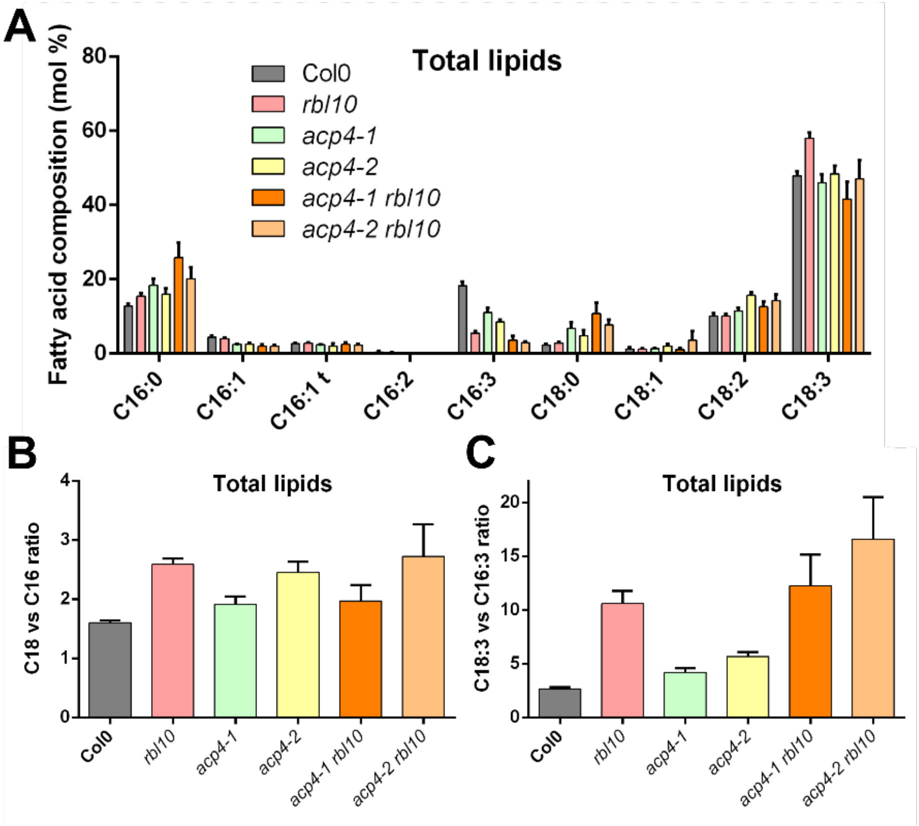

Supplemental Figure S1. Lipid characterization of leaves from the *acp4*, *rbl10*, and *acp4 rbl10* mutant plants. (A) Fatty acid composition of total lipids. (B) Ratio of total C18 to C16 fatty acids in total lipids. (C) Ratio of C18:3 to C16:3 fatty acids in total lipids. Data represent the mean ± S.D., n = 6 of independent lines. Fatty acid composition is presented as a mole percentage of total fatty methyl esters.

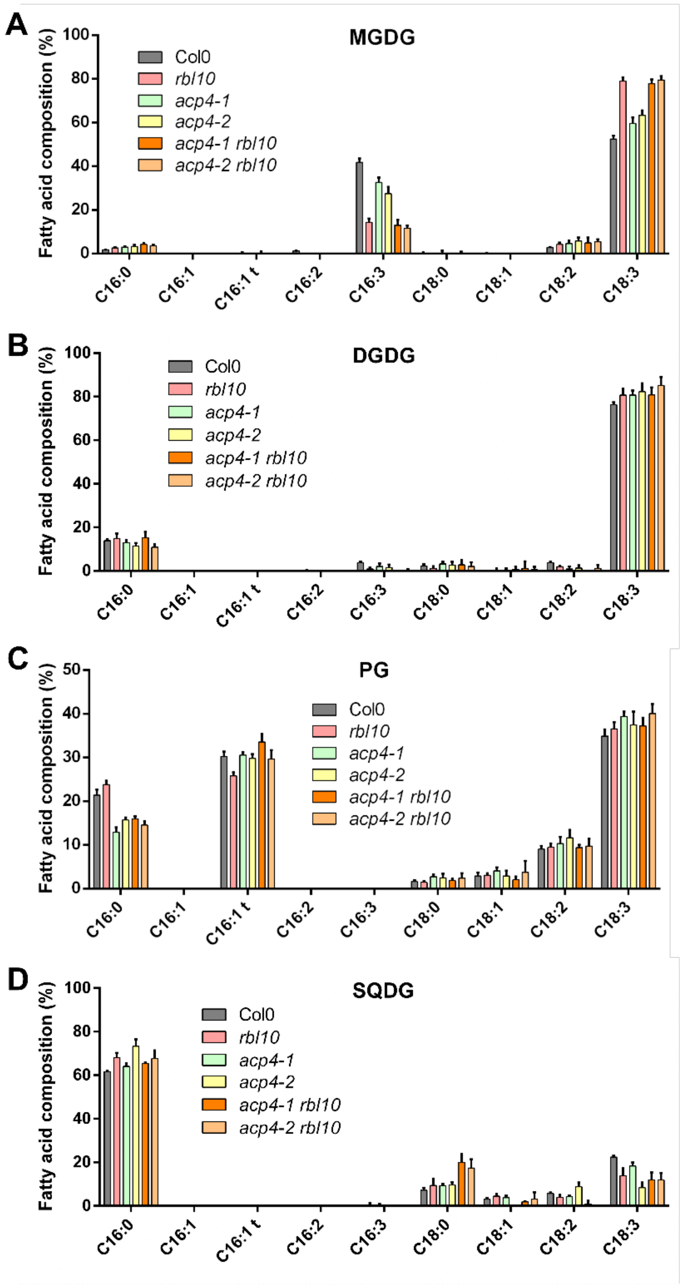

Supplemental Figure S2. Fatty acid composition of different lipid classes of leaves from the *acp4*, *rbl10*, and *acp4 rbl10* mutant plants. (A) Fatty acid composition of MGDG. (B) Fatty acid composition of DGDG. (C) Fatty acid composition of PG. (D) Fatty acid composition of SQDG. Data represent the mean ± S.D., n = 6 of independent lines. Fatty acid composition is presented as a mole percentage of total fatty methyl esters. DGDG, digalactosyldiacylglycerol; MGDG, monogalactosyldiacylglycerol; PG, phosphatidylglycerol; SQDG, sulfoquinovosyldiacylglycerol.

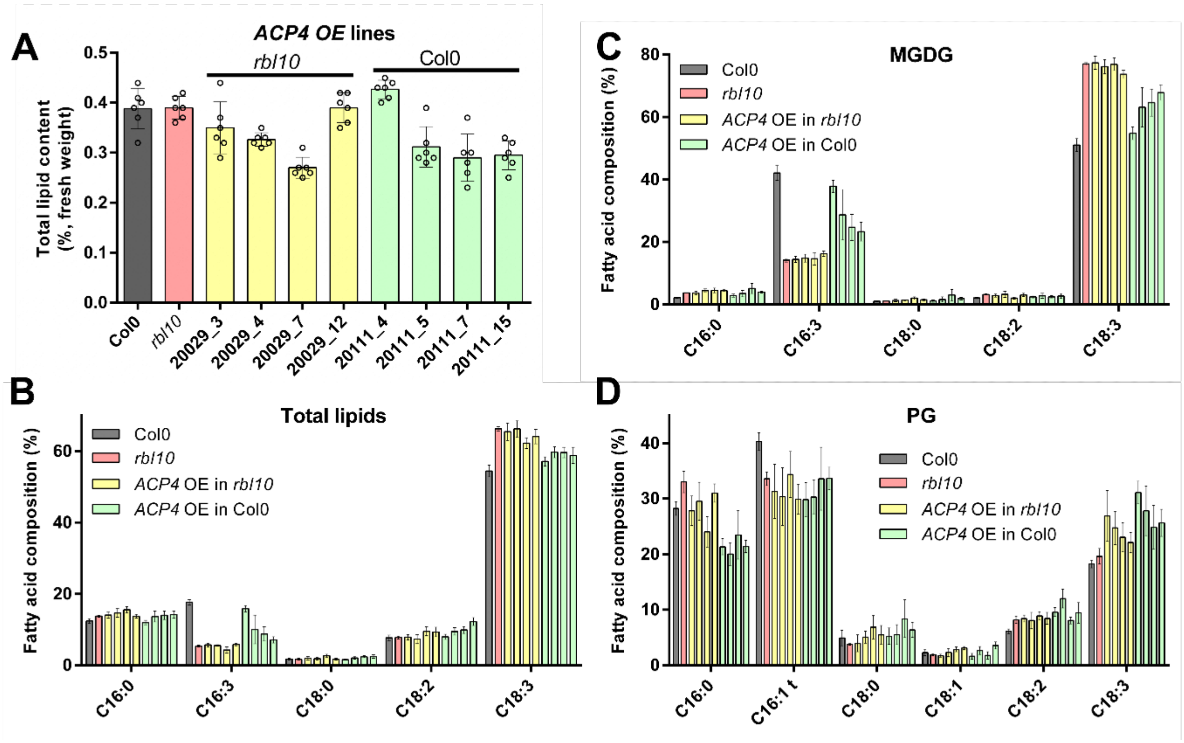

Supplemental Figure S3. Lipid characterization of leaves from the *ACP4* over-expression lines in Col-0 and *rbl10* mutant backgrounds. (A) Total lipid content in the leaves of Col-0 and *rbl10* lines over-expressing *ACP4* under the *CaMV 35S* promoter. (B) Fatty acid composition of total leaf lipids. (C) Fatty acid composition of leaf MGDG. (D) Fatty acid composition of leaf PG. Data represent the mean ± S.D., n = 6 of independent lines. Fatty acid composition is presented as a mole percentage of total fatty methyl esters. MGDG, monogalactosyldiacylglycerol; PG, phosphatidylglycerol.

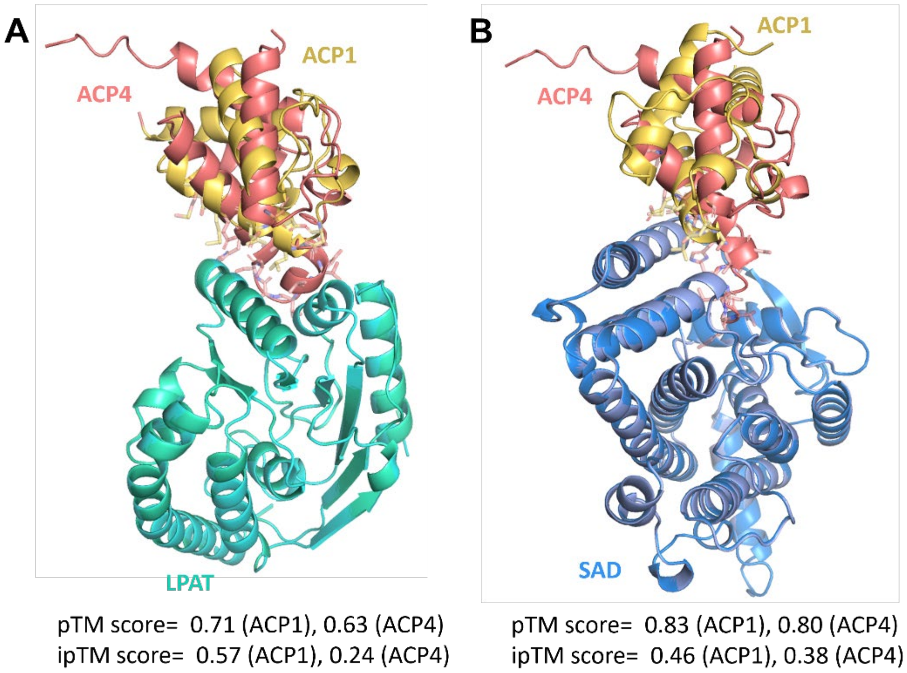

Supplemental Figure S4. Predicted protein–protein interaction structures of ACP1 or ACP4 with LPAT or SAD using AlphaFold2. (A) LPAT. (B) SAD. The intrinsic model accuracy estimates for the AlphaFold model (pTM) and AlphaFold-Multimer model (ipTM) are shown.

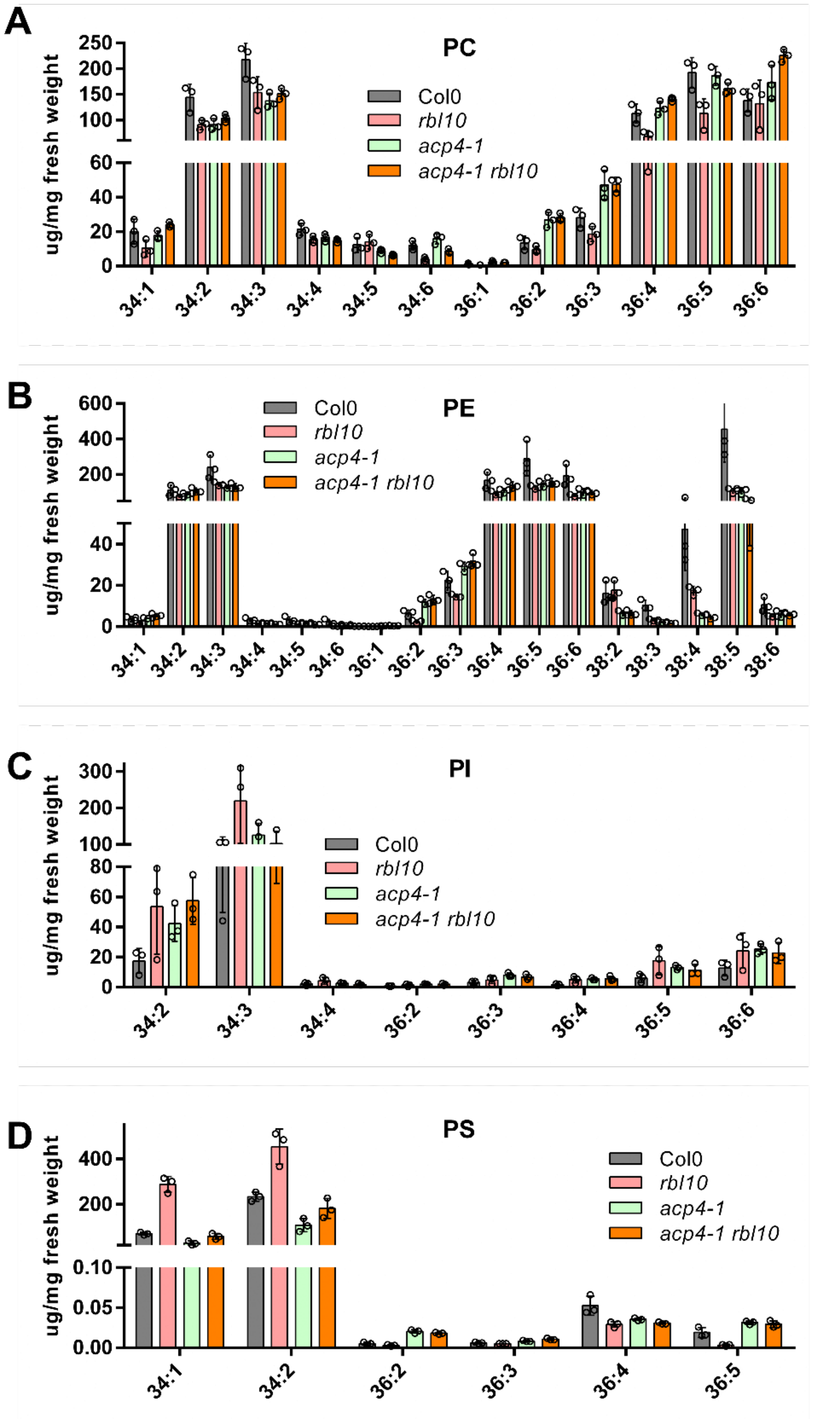

Supplemental Figure S5. Molecular species of phospholipids of leaves from the *acp4*, *rbl10*, and *acp4 rbl10* mutant plants. (A) Molecular species of PC. (B) Molecular species of PE. (C) Molecular species of PI. (D) Molecular species of PS. Data represent the mean ± S.D., n = 3. PC, phosphatidylcholine; PE, phosphatidylethanolamine; PI, phosphatidylinositol; PS, phosphatidylserine.
